# *HvEXPB7*, a novel β-expansin gene revealed by the root hair transcriptome of Tibetan wild barley, improves root hair growth under drought stress

**DOI:** 10.1093/jxb/erv436

**Published:** 2015-09-28

**Authors:** Xiaoyan He, Jianbin Zeng, Fangbin Cao, Imrul Mosaddek Ahmed, Guoping Zhang, Eva Vincze, Feibo Wu

**Affiliations:** ^1^Department of Agronomy, College of Agriculture and Biotechnology, Zijingang Campus, Zhejiang University, Hangzhou 310058, PR China; ^2^Department of Molecular Biology and Genetics, University of Aarhus, Fosøgsvej 1, DK-4200 Slagelse, Denmark

**Keywords:** BSMV-VIGS, drought, RNA-Seq, root hair, Tibetan wild barley, β-expansin gene (*HvEXPB7*).

## Abstract

Tibetan wild barley is a treasure trove of useful genes for crop improvement including abiotic stress tolerance, like drought. Root hair of single-celled structures plays an important role in water and nutrition uptake. Polyethylene-glycol-induced drought stress hydroponic/petri-dish experiments were performed, where root hair morphology and transcriptional characteristics of two contrasting Tibetan wild barley genotypes (drought-tolerant XZ5 and drought-sensitive XZ54) and drought-tolerant cv. Tadmor were compared. Drought-induced root hair growth was only observed in XZ5. Thirty-six drought tolerance-associated genes were identified in XZ5, including 16 genes specifically highly expressed in XZ5 but not Tadmor under drought. The full length cDNA of a novel β-expansin gene (*HvEXPB7*), being the unique root hair development related gene in the identified genes, was cloned. The sequence comparison indicated that *HvEXPB7* carried both DPBB_1 and Pollon_allerg_1 domains. *HvEXPB7* is predominantly expressed in roots. Subcellular localization verified that HvEXPB7 is located in the plasma membrane. Barley stripe mosaic virus induced gene silencing (BSMV-VIGS) of *HvEXPB7* led to severely suppressed root hairs both under control and drought conditions, and significantly reduced K uptake. These findings highlight and confer the significance of *HvEXPB7* in root hair growth under drought stress in XZ5, and provide a novel insight into the genetic basis for drought tolerance in Tibetan wild barley.

## Highlight

A novel root hair development related gene, *HvEXPB7*, was identified and cloned from the identified drought tolerance-associated genes. BSMV-VIGS of *HvEXPB7* confirmed that this gene was involved in root hair growth under drought.

## Introduction

Drought is one of the most severe abiotic factors affecting crop productivity worldwide. A cost-effective solution for sustainable crop production in water-limiting areas is the development of crop cultivars with high drought tolerance (Ahmed *et al.*, 2013). Thus the identification of drought-tolerant germplasm and an understanding of the tolerance mechanisms are quite imperative. When plants are exposed to drought stress, their roots are one of the primary sites for receiving stress signals that initiates a cascade of gene expression for responses to drought. Gene expression patterns in roots under drought stress have been studied in a variety of plants, for example *Arabidopsis* (Dinneny *et al.*, 2008), common bean (Micheletto *et al.*, 2007), sunflower (Liu and Baird, 2003) and rice (Wang *et al.*, 2007). However, little is known about gene expression patterns under water deficit in root hairs of genotypes differing in drought tolerance.

Root hairs in plants form an important and large surface area for efficient absorption of water and mineral nutrients. Working on the single cell of a root hair rather than an entire tissue could avoid potential dilution effects, enabling a more sensitive and accurate depiction of the plant cellular response to a specific environmental stress. The mechanisms of root hair development have been studied extensively in *Arabidopsis* (Libault *et al.*, 2010). However, the molecular information on root hair development in monocot species is limited to the identification of a single functional gene in mutants of maize (Hochholdinger *et al.*, 2008), rice (You *et al.*, 2009) and barley (Kwasniewski and Szarejko, 2006; Kwasniewski *et al.*, 2010; Bielach *et al.*, 2012). Recently, high-throughput cDNA sequencing provides an efficient method for characterizing gene expression profiling in plants. However, no information is available regarding transcriptome changes of root hairs in response to drought stress.

Virus-induced gene silencing (VIGS) is a powerful functional genomics tool for the rapid targeted down-regulation of host plant genes. Barley stripe mosaic virus (BSMV) has recently been developed as a VIGS vector for monocots (Lee *et al.*, 2012) and generates a robust silencing response in barley and wheat (Kang *et al.*, 2013). Cell wall loosening, potentially mediated by cell wall-loosening expansin proteins (EXPs), is essential for the initiation and growth of root hairs (Cho and Cosgrove, 2002; Yu *et al.*, 2011). There are four EXP families: EXPA, EXPB, EXLA and EXLB. EXPA proteins function more efficiently on dicot cell walls, while EXPB proteins show specificity for grass cell walls regardless of their dicot or grass origin (Sampedro *et al.*, 2006). Two root hair specific *cis*-elements (RHE)-containing *Arabidopsis* EXPA genes (*AtEXPA7* and *AtEXPA18*) are expressed specifically in root hair cells immediately prior to root hair initiation (Kim *et al.*, 2006). An EXPB gene of rice (*OsEXPB5*), which contains putative RHE sequences in its promoter, is shown to direct root-hair-specific gene expression in both *Arabidopsis* and rice roots (Won *et al.*, 2010). Disruption of these EXP genes could be used to examine their function.

Barley (*Hordeum valgare* L.), the fourth most important cereal in the world, has been considered an ideal model for genetic and physiological studies. Compared to other cereals, barley is relatively high in drought tolerance, so is the most suitable plant in drought-related research, and the most promising sources of drought-related genes. However, modern barley cultivars have become narrower in genetic diversity, resulting in more sensitivity to abiotic and biotic stresses. In contrast, wild barley is much richer in genetic diversity, and can offer elite germplasm for crop improvement. Tibetan annual wild barley from the Qinghai-Tibet Plateau is regarded as one of the progenitors of cultivated barley and is rich in genetic diversity (Dai *et al.*, 2014).

Two contrasting Tibetan barley genotypes, XZ5 (drought tolerant) and XZ54 (drought sensitive), with marked different root hair development in response to drought stress were successfully identified by Zhao *et al.*, 2010. Therefore the question arises whether any unique strategy of drought tolerance exists on root hair transcriptome in the wild barley compared with cultivated barley. Thus, the research priority of the present study is to gain a deeper understanding of the molecular mechanisms occurring in root hair in response to drought. Large-scale transcriptome sequencing of two wild barley (XZ5, drought tolerant; XZ54, drought sensitive) and one cultivated barley (Tadmor, drought tolerant) genotypes were performed under drought and normal conditions using Illumina RNA-sequencing technology. As a result, a novel β-expansin gene (*HvEXPB7*) was identified and cloned. It is a unique root hair development-related gene, and differently expressed under drought between XZ5 and Tadmor. Moreover, the function of *HvEXPB7* in root hair development was verified by BSMV-VIGS. The results obtained will help in understanding the mechanisms of drought tolerance and the breeding of drought-tolerant barley cultivars.

## Materials and methods

### Hydroponic experiment for root hair morphology observation

The two Tibetan wild barley (*Hordeum vulgare* L. ssp. *spontaneum*) genotypes, XZ5 (drought tolerant) and XZ54 (drought sensitive) (Zhao *et al.*, 2010), and a drought-tolerant cv. Tadmor (Forster *et al.*, 2004) were used in this study. Healthy seeds were surface-sterilized in 2% H_2_O_2_ for 30min, rinsed thoroughly with distilled water, and then germinated on sterilized moist filter papers in an incubator (22/18°C, day/night) for 7 d. The uniform seedlings were selected and transplanted to a 5 l black plastic bucket filled with 4.5 l basal nutrient solution (BNS). The composition of BNS was described in Zhao *et al.* (2010). Each container was covered with a polystyrol plate with nine evenly spaced holes (two plants per hole) and placed in a greenhouse. After 7 days’ growth, the solution was renewed and the corresponding containers were supplemented with 20% (w/v) polyethylene glycol (PEG 6000) to form about −0.84MPa osmotic potential stress, which was determined by a Vapro vapour pressure osmometer (Wescor, USA). Control plants were maintained in BNS without adding PEG. The experiment was laid in a split-plot design with treatment as the main plot and genotype as the sub-plot, and there were six replicates for each treatment. The solution pH was adjusted to 5.8±0.1 with NaOH or HCl as required and continuously aerated with pumps throughout the experiment. Tap roots (five replicates, each containing six plants) for observing root hair morphology were taken after 1, 3 and 5 days’ treatment, respectively, and then were observed by stereomicroscope (SZX12, Olympus, Japan) and scanning electron microscope (SEM) (JSM-IT300 JEOL, Japan).

### Petri dish experiment for root hair isolation

For the Illumina sequencing analyses, germinated seeds of XZ5, XZ54 and Tadmor were placed on plates containing 1/2 MS medium and 0.8% agar (pH 5.8±0.1) with (drought) or without (control) 20% PEG 6000 (Verslues *et al.*, 2006). All plates were grown vertically in a growth chamber. The roots of at least 200 barley seedlings for each treatment were harvested every 5h from 3−5-day-old seedlings under drought or control conditions to minimize the effect of root hair development stages. The roots were frozen in liquid nitrogen, and the root hairs were scraped off with a spattle cooled in liquid nitrogen (Supplementary Fig. S1). The roots and spattle were kept continuously in liquid nitrogen during the operation. Once a sufficient amount of root hairs was collected, the liquid nitrogen containing the root hairs was poured into a 50ml plastic tube placed on ice. The liquid nitrogen was allowed to evaporate until 10ml remained, and then the tube was closed with a perforated cap and stored at −80°C.

### Total RNA extraction, RNA-Seq library construction and Illumina sequencing

Total RNA isolation was carried out according to the instructions of the RNeasy Plant Mini Kit (QIAGEN, Germany). mRNA enrichment was obtained from the total RNA using poly-T oligonucleotide-attached magnetic beads. Then the mRNA was randomly broken into fragments. cDNA was synthesized using reverse transcriptase combined with random primers and with adapters ligated at both ends. With those adapter sequences, the double-stranded DNA fragments were selectively amplified and enriched. We used Qubit fluorometric quantification by Agilent 2100 and real-time quantitative PCR for accurate quantification before sequencing.

The PCR products were loaded onto the Illumina HiSeq2000 platform for 2×100bp paired-end sequencing. Then, the RNA-Seq reads were generated via the Illumina data processing pipeline (Version 1.8). To obtain clean data, the raw reads were trimmed by removing N ratio reads greater than 10%, adaptor sequences and low quality bases at the 3ʹ ends. All the clean reads were considered for further analysis. The barley genome sequence and annotation data were downloaded, and TopHat v. 2.0.5 was adopted to align the RNA-Seq clean reads to the barley reference genome. Then, the clean reads were mapped against the reference genome and statistically analysed.

Uniquely mapped reads were used for gene expression analysis, with the expression level of each gene calculated by quantifying the number of reads. Gene expression counts were normalized by a variation of the FPKM (fragments per kilobase of exon per million fragments mapped) method (Ji *et al.*, 2013). To identify differentially expressed genes (DEGs) between two different samples, the Cufflinks software was employed to output the T-statistic and the *P*-values for each gene. The false discovery rate (FDR) was used to determine the threshold of *P*-values for multiple test and analysis by Cuffdiff (part of the Cufflinks suite of analysis tools). An FDR<0.05 and a relative value of the log_2_ ratio ≥2 provided the significance thresholds for gene expression differences.

### Gene ontology (GO) annotation and functional classification

The genes were annotated using the publicly available Blast2GO package v. 2.7.0. First, the blastx was run through QBlast against the NCBI non-redundant (nr) protein database. Then, the mapping step was run, and then the annotation step was performed using an e-value threshold of 1.0e-6. In addition, a comparison against the InterPro domain database was used to increase the number of annotated sequences using InterProScan, and GO-slim was used to enhance the recall rate. The Kyoto Encyclopedia of Genes and Genomes (KEGG) pathway online database (http://www.genome.jp/kegg/pathway.html) was used for KEGG analysis.

### Quantitative real-time PCR validation

The petri dish experiment for root hair isolation was carried out once again using XZ5, XZ54 and Tadmor under control and 20% PEG 6000-induced drought conditions and the total RNA was isolated as described above. First-strand cDNA synthesis was performed with 1 μg of total RNA using the PrimeScipt^TM^ RT reagent Kit with gDNA Eraser (Takara, Japan), according to the manufacturer’s instructions. The transcriptional profiles of five genes were analysed by quantitative real-time PCR (qRT-PCR) using the SYBR Green Supermix (Bio-Rad, USA) and a CFX96 system (Bio-Rad, USA). The expression levels of the tissue-enriched transcripts were normalized using an endogenous actin control. Each set of experiments was repeated three times, and the 2^-ΔΔCq^ relative quantification method was used to evaluate quantitative variation. The primer sets used in this study to validate the RNA-Seq results are provided in Supplementary Table S1.

### Cloning the full-length cDNA of *HvEXPB7*

Based on the sequence of the *HvEXPB7* cDNA fragment isolated from the root hair transcriptome, the gene-specific primers GSPF and GSPR, together with the adaptor primer UPM (Supplementary Table S2), were designed to obtain the unknown sequence at the 3ʹ and 5ʹ ends by RACE-PCR. RACE was performed using a SMARTer RACE cDNA Amplification Kit (Clontech, USA), according to the manufacturer’s instructions. Then, the full-length cDNA and DNA sequences were amplified using the primers of EXPB7F and EXPB7R (Supplementary Table S2). The resultant PCR products were cloned into the pMD18-T vector (Takara, Japan) and then sequenced.

### Subcellular and tissue localization of *HvEXPB7*

The ORF of *HvEXPB7* was directly amplified from the full-length cDNA using the primers of EXPB7-35S:sGFP-F and EXPB7-35S:sGFP-R (Supplementary Table S2) and then cloned into the binary vector pCAMBIA 1300 containing a CaMV 35S promoter::GFP cassette to create HvEXPB7-GFP fusion protein. The 35S:HvEXPB7-GFP fusion construct was introduced into the *Agrobacterium tumefaciens* strain EHA105 using a freeze-thaw method. *Agrobacterium*-mediated infection of *Nicotiana benthamiana* was performed as described previously (Xu *et al.*, 2014). The above vector was also transiently expressed in onion epidermal cells using the Biolistic PDS-1000/He system (Bio-Rad, USA) with 1100 psi rupture discs under a vacuum of 27 inch of Hg, according to the manufacturer’s directions as described by Varagona *et al.* (1992). Green fluorescent protein (GFP) fluorescence was imaged using the LSM780 laser scanning system (Carl Zeiss, Germany).

Tissue-specific expression analysis of *HvEXPB7* from XZ5 was carried out in three replicates using seedlings grown in BNS for 21 d and grains (five grains for each replicate). RNA extraction, reverse transcription and RT-PCR were conducted as described in above, and the primers are shown in Supplementary Table S2.

### Construction of BSMV-derived vectors, *in vitro* transcription, BSMV inoculation and gene function evaluation

BSMV has a positive sense, single stranded, tripartite RNA genome, i.e. RNAs α, β and γ. The γb ORF in the RNAγ cDNA clone can be manipulated to accommodate the exon fragment of the target gene. RT-PCR with oligonucleotide primers containing *Nhe*I sites (Supplementary Table S2) was performed to obtain a 286bp cDNA fragment of barley phytoene desaturase gene (*HvPDS*) and a 258bp cDNA fragment of the β-expansin gene (*HvEXPB7*). These two gene sequences were reversely inserted into the RNAγ cDNA strand to prepare cDNA clones of BSMV:HvPDS and BSMV:HvEXPB7 for the appropriate gene silencing in XZ5.

For the *in vitro* transcription reactions, RNAα, RNAγ and RNAγ-derivative clones were linearized with *Mlu*I and RNAβ with *Spe*I. RNA synthesis was carried out using the RiboMAX™ Large Scale RNA Production System-T7 kit (Promega, USA), according to the manufacturer’s instructions. The RNAα, RNAβ and RNAγ (or its derivative) transcripts were mixed in a 1:1:1 ratio, which was subsequently diluted with three volumes of RNase-free water. An equal volume of 2× GKP buffer (Petty *et al.*, 1989) was added to the diluted transcript mixture for subsequent inoculations.

The inoculation was performed on the second leaf of the XZ5 plants at the two-leaf stage and was accomplished by gently rubbing the leaf surface with a gloved finger; 8 μl was used for each seedling. For mock inoculation on wild-type (WT) control seedlings, 8 μl of a mixture of empty vector transcripts and GKP buffer in a 1:1 ratio was used for each seedling. After inoculation, the seedlings were fog-sprayed with nuclease-free water and covered with plastic film to maintain high humidity for 3 d. All the inoculated and mock-inoculated plants were maintained in a greenhouse (22/18°C, day/night) and checked for virus symptoms at regular intervals.

For the BSMV:HvPDS VIGS, leaf samples were chosen based on observed phenotype, and a picture of the leaf was taken with a camera (EOS 7D, Canon, Japan). To confirm the VIGS effectiveness and function of *HvEXPB7* in XZ5, four independent sets of treatments were performed: mock-inoculated with BSMV:γ; mock-inoculated with BSMV:γ and treated with 20% PEG 6000; BSMV:HvEXPB7-inoculated seedlings; and BSMV:HvEXPB7-inoculated seedlings treated with 20% PEG 6000. Every treatment was repeated five times, and each replicate contained five plants. The root hair morphology was observed by stereomicroscope (SZX12, Olympus, Japan). In addition, the transcript levels of *HvPDS* and *HvEXPB7* were also measured using quantitative RT-PCR and semi-quantitative RT-PCR, respectively.

### Determination of root potassium (K^+^) concentration

The roots were thoroughly rinsed with tap water and dried at 80°C for 72h until their weight remained constant. Dry roots were ground into powder, and were prepared for K^+^ content determination using Inductively Couple Plasma-Optical Emission Spectroscopy (ICP-OES) (Optima 8000 DV, PerkinElmer, USA).

### Statistical analysis

Statistical analysis was performed with data processing system (DPS) statistical software package. One-way ANOVA followed by the Duncan’s Multiple Range Test (DMRT) were used to evaluate treatment effects at significance level of *P*<0.05.

## Results

### Root hair growth of the three barley genotypes is affected by drought stress

**Fig. 1.**
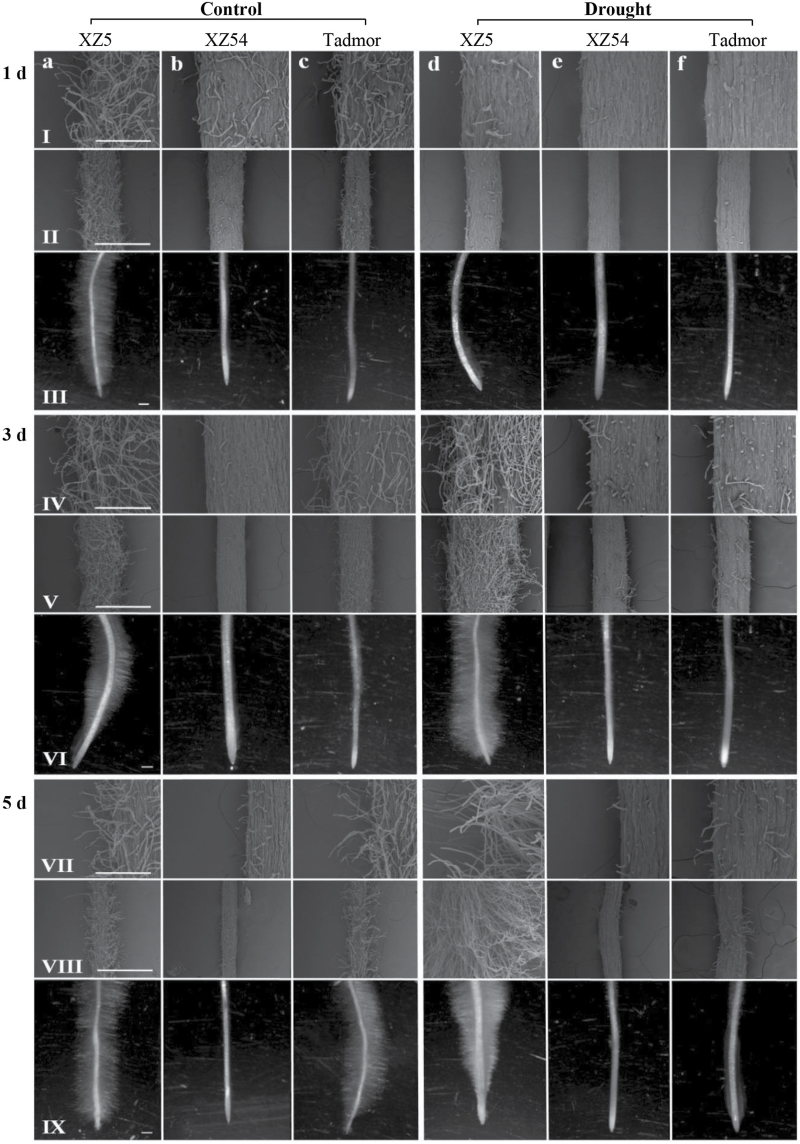
Root hair morphology of XZ5, XZ54 and cv. Tadmor under control (columns a, b, c) and drought conditions (columns d, e, f). Root hair stereomicroscope (lines III, VI, IX) and scanning electron microscope (SEM) (lines I, II, IV, V, VII, VIII) images of XZ5, XZ54 and Tadmor were observed after 1 (line I, II, III), 3 (IV, V, VI), and 5 (VII, VIII, IX) days drought stress. Scale bars: Stereoscope, 1mm (line III, VI, IX); SEM, 300 μm (line II, V, VIII) and 100 μm (line I, IV, VII). Figure is representative from five different experiments.

The effect of drought on root hair growth was observed via stereomicroscope and SEM. As shown in Fig. 1, XZ5 exhibits significantly more root hairs than XZ54 and cv. Tadmor on day 1 and 3 under control and PEG 6000-induced drought stress. On day 5, XZ5 had much more and longer root hairs in the drought treatment than in the control, while XZ54 and Tadmor had less and even shorter root hairs under drought in comparison with the control.

### Gene expression in the root hairs of the three barley genotypes is affected by drought stress

Deep sequencing of six cDNA root hair libraries produced 165 459 968 clean reads with a length of 100bp each (Supplementary Table S3). Of the 24−29 million clean reads from each library, 59−76% were mapped to unique locations, whereas 12−26% were mapped to multiple locations in the genome. Meanwhile, the number of expressing genes found in each sample ranged from 42 570 to 46 383, thus providing large datasets for further expression profile analysis.

**Fig. 2.**
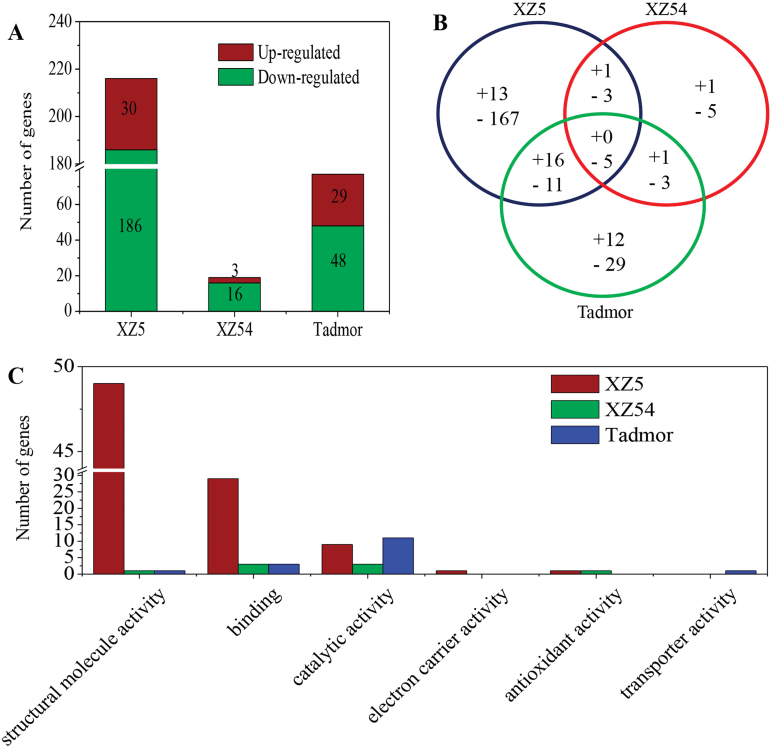
Distribution of root hair DEGs in XZ5, XZ54 and Tadmor in response to drought stress and their function prediction. (A) Histogram of up- and down-regulated gene number of the three barely genotypes. (B) Venn diagram of up- and down-regulated genes (+, up regulated; -, down regulated). (C) Molecular function classification of all DEGs according to Blast2GO and non-redundant (nr) protein database.

Overall, gene expression profiles of the three barley genotypes were significantly altered after 3−5 d in PEG compared with the control. Drought stress induced differential expression of 266 genes in root hairs, with a log_2_ (fold change) of at least 2.0 (FDR<0.05). Among these DEGs, 30 (186), 3 (16) and 29 (48) were up-regulated (down-regulated) in XZ5, XZ54 and Tadmor, respectively (Fig. 2A, B). GO enrichment analysis of these DEGs showed significant differences among the three barley genotypes. The 266 DEGs belonged to six important regulatory functional classes (Fig. 2C); among them, structural molecule activity (GO: 0005198), binding (GO: 0005488) and catalytic activity (GO: 0003824) were the main categories.

**Fig. 3.**
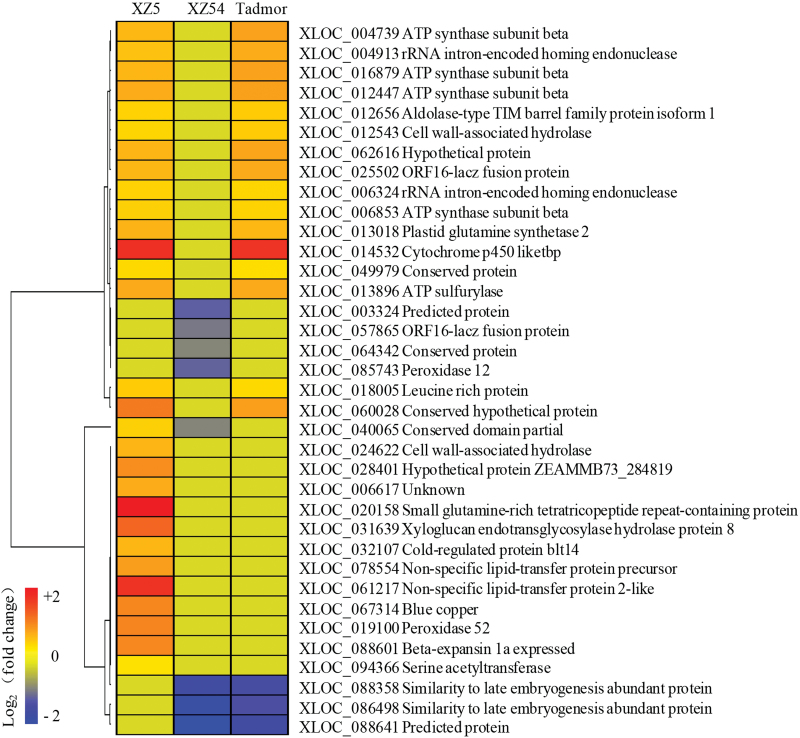
Hierarchical cluster analysis of 36 root hair DEGs. The expression of the DEGs was up-regulated in XZ5 but suppressed/unchanged in XZ54, or unchanged in XZ5 but suppressed in XZ54 (drought vs. control). Hierarchical clustering of DEGs was displayed by Pearson correlation and pairwise average-linkage as a measurement of similarity. Functional annotation was done according to Blast2GO and non-redundant (nr) protein database.

The 266 DEGs could be clustered into five categories according to similarities in their expression profiles (Supplementary Fig. S2; Supplementary Tables S4−8). Among the 36 genes that were up-regulated in XZ5 but down-regulated/unchanged in XZ54 or unchanged in XZ5 but down-regulated in XZ54, 25% were related to stress and defence, and 11% represented the functions related to root hair development or cell wall modification (Fig. 3; Supplementary Fig. S2A; Supplementary Table S4). Of the 180 genes that were up-regulated in XZ54 but down-regulated/unchanged in XZ5 or unchanged in XZ54 but down-regulated in XZ5, except for unknown classified and non-function sections, the largest portion (44%) was related to protein synthesis, followed by transcription (15%) (Supplementary Fig. S2B; Supplementary Table S5). The gene that was up-regulated in both XZ5 and XZ54 was a cortical cell-delineating gene (Supplementary Fig. S2C; Supplementary Table S6). In addition, the eight genes that were down-regulated in both XZ5 and XZ54 were mainly involved in unknown classified and non-function sections, followed by 1 histone h2a and 1 o-methyltransferase (Supplementary Fig. S2D; Supplementary Table S7). Furthermore, of the 41 genes that were only up- or down-regulated in Tadmor, 29% were mainly involved in metabolism, followed by cell growth (24%) (Supplementary Fig. S2E; Supplementary Table S8).

### Drought tolerance-associated DEGs in the drought-tolerant wild barley genotype XZ5

The most relevant group related to drought tolerance were the 36 genes which were up-regulated in XZ5 and down-regulated or unchanged in the XZ54 or unchanged in XZ5 but down-regulated in XZ54 (Supplementary Fig. S2A; Supplementary Table S4). Hierarchical clustering of the 36 DEGs revealed two major clusters (Fig. 3). The gene expression patterns of XZ5 were the same as that of Tadmor for one of those two clusters, including 16 genes that were up-regulated in XZ5 and Tadmor simultaneously but unaltered/down-regulated in XZ54, and four genes that were unaltered in XZ5 and Tadmor but down-regulated in XZ54 under drought stress. For the other cluster, the gene expression patterns differed between XZ5 and Tadmor, including 12 genes that were up-regulated in XZ5 but unaltered-regulated in XZ54 and Tadmor and three genes that were unaltered in XZ5 but down-regulated in XZ54 and Tadmor, and also one gene up-regulated in XZ5 but down-regulated in XZ54 and unaltered in Tadmor under drought stress.

Four genes, XLOC_013018, XLOC_013896, XLOC_ 019100 and XLOC_012656, encoding enzymes of plastid glutamine synthetase 2, ATP sulfurylase, peroxidase 52 and aldolase-type TIM barrel family protein isoform 1, respectively, were assigned to 11 KEGG pathways, using KEGG pathway enrichment, which was mainly involved in arginine, proline, glutathione, glyoxylate and phenylalanine metabolism (Supplementary Table S9).

**Fig. 4.**
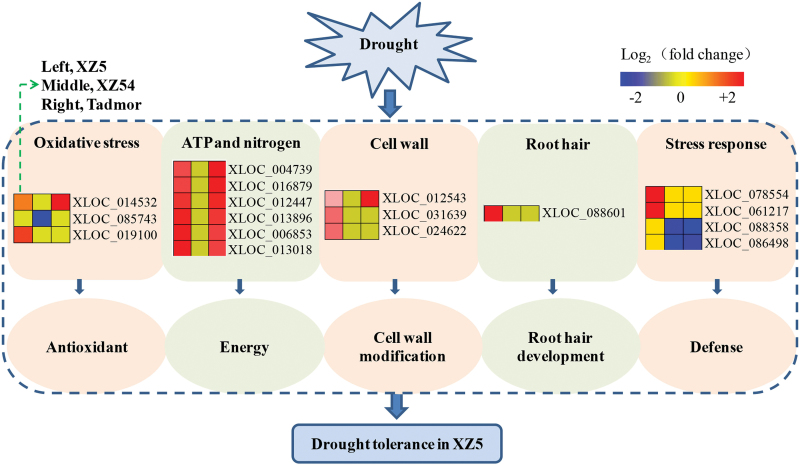
A predicted root hair DEGs-based drought-tolerance-associated model in Tibetan wild barley XZ5 in response and adaptation to drought stress. XLOC_014532: Cytochrome p450; XLOC_085743: Peroxidase 12; XLOC_019100: Peroxidase 52; XLOC_004739: ATP synthase subunit beta; XLOC_016879: ATP synthase subunit beta; XLOC_012447: ATP synthase subunit beta; XLOC_013896: ATP sulfurylase; XLOC_006853: ATP synthase subunit beta; XLOC_013018: Plastid glutamine synthetase 2; XLOC_012543: Cell-wall associated hydrolase; XLOC_031639: XTH8; XLOC_024622: Cell-wall associated hydrolase; XLOC_088601: Beta-expansin; XLOC_078554: *nsLTP*; XLOC_061217: *nsLTP*; XLOC_088358: *LEA*, XLOC_086498: *LEA*.

Accordingly, an integrated schematic diagram was proposed based on the 17 identified drought tolerance-associated genes, including 4 ATP synthase subunit beta, 1 ATP sulfurylase, 2 peroxidase, 1 cytochrome P450, 2 cell-wall-associated hydrolase, 1 xyloglucan endotransglycosylase hydrolase, 1 β-expansin, 2 late embryogenesis abundant (LEA)-like genes and 2 nonspecific lipid transfer protein-like genes (Fig. 4).

### The differential expression genes are verified via qRT-PCR

To verify the RNA-Seq data, five DEGs that expressed more highly in XZ5 than in the other two genotypes (drought vs. control) were selected for qRT-PCR validation. The five DEG were the β-expansin gene (XLOC_088601), non-specific lipid-transfer protein precursor (XLOC_078554), LEA gene (XLOC_086498), blue copper (XLOC_067314) and an unknown gene (XLOC_006617). The primers targeting these genes are listed in Supplementary Table S1. The expression patterns determined by both qRT-PCR and RNA-Seq were consistent for all five genes (Supplementary Fig. S3), suggesting that our RNA-Seq analyses were reliable. These representative genes showed consistently higher expression levels in XZ5 compared to XZ54 and Tadmor. For instance, the average relative expression of the β-expansin gene (being the unique root hair development related gene in the identified genes) in XZ5 was 5.6, and it was 4- and 7-fold higher than the relative expression level in XZ54 and Tadmor under drought stress (Supplementary Fig. S3).

### Isolation, cloning and sequencing of *HvEXPB7* in root hair of barley

**Fig. 5.**
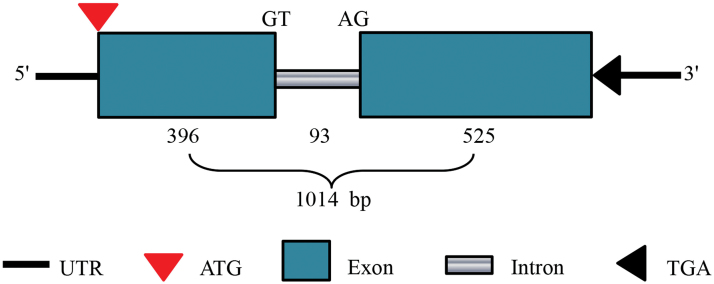
Gene structure of *HvEXPB7*. UTR is the untranslated region.

**Fig. 6.**
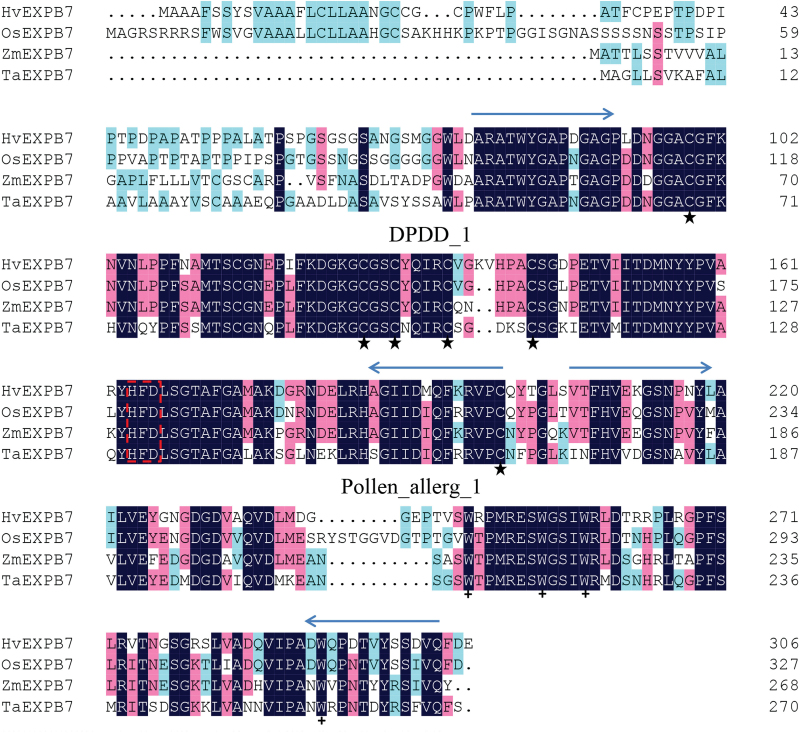
Alignment analysis of amino acid sequences of HvEXPB7 with three other plant species. OsEXPB7 is from rice, ZmEXPB7 is from maize and TaEXPB7 is from wheat. The dark blue represents 100% identity, the pink represents 75% identity, the blue-green represents 50% identity, as defined by ClustalX. Blue arrows point out the two conserved domains: DPDD_1 domain and Pollen_allerg_1 domain. (★) mark the six C (cysteine) residues. (**+**) show four W (tryptophan) residues in the C-terminal region. The red box marks the HFD (His-Phe-Asp) motif in the centre of the sequence.

**Fig. 7.**
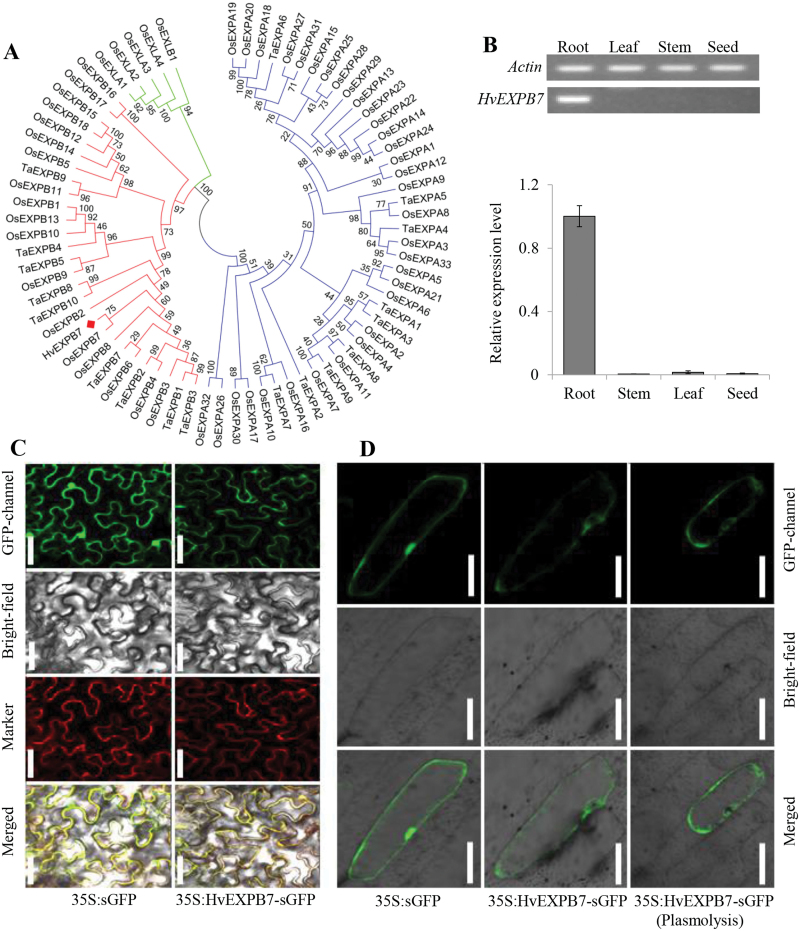
Phylogenetic tree, tissue expression pattern and subcellular localization of *HvEXPB7*. (A) Phylogenetic tree of EXPs proteins in three representative genomes of grass plants. The alignment protein sequences are listed in Supplementary Text S2. (B) RT-PCR analysis of the relative transcript levels of *HvEXPB7* in different tissues of XZ5. (C) GFP and HvEXPB7-sGFP fusion protein transiently expressed in tobacco. Microscopic images from top: green fluorescence of the constructs, bright-field, red fluorescence of pm–rb CD3-1008 (plasma membrane-localized marker) and merged microscope images. (D) Transient expression of the GFP and HvEXPB7-sGFP fusion protein in onion epidermis cells. Microscopic images from top: green fluorescence of HvEXPB7-sGFP, bright-field and merged microscope images. Scale bars, 50 μm.

Based on the cDNA fragment sequence of XLOC_088601 from the RNA-Seq results, the full-length cDNA of a new β-expansin gene (*HvEXPB7*, GenBank: KR732966 will be released on 1 June 2016) was obtained by RACE-PCR (Supplementary Fig. S4). This *HvEXPB7* is a 1278bp cDNA, and its encoding protein consists of 306 amino acid residues with a predicted molecular weight of 32kDa and the theoretical pI 4.79. This gene contained a 93bp intron in the middle of the DNA sequence (Fig. 5). Deduced amino acid sequence analysis of *HvEXPB7* by SMART revealed two functional domains: the DPDD_1 domain and the Pollen_allerg_1 domain, which work together during cell wall loosening (Cosgrove, 2015). Comparative analysis of the HvEXPB7 protein sequence with the known EXPBs in plants using the Clustal X method indicated that conserved motifs were shared among all EXPBs (Fig. 6; Supplementary Text S1), including six C (cysteine) residues in the N-terminal region forming three disulfide bonds, an HFD (His-Phe-Asp) motif in the centre, and four W (tryptophan) residues in the putative cellulose-binding domain in the C-terminal region. Furthermore, using the MEGA 5 program with the neighbor-joining algorithm analysis method, a phylogenetic tree was constructed of the protein sequences of HvEXPB7, 9 EXPAs and 9 EXPBs from wheat (*Triticum aestivum*), 33 EXPAs and 18 EXPBs from rice (*Oryza sativa*), plus four OsEXLAs and one OsEXLB (Fig. 7A; Supplementary Text S2). The phylogenetic results indicated that HvEXPB7 is most closely related to OsEXPB7.

According to the reference DNA sequence of Morex from the IPK Barley Blast Server, the homologous promoter sequences, upstream from the start codon (ATG) of *HvEXPB7*, were isolated from XZ5, XZ54 and Tadmor (GenBank: KT000616, KT000617 and KT000618 will be released on 1 June 2016), with lengths of 2503bp, 2501bp and 2500bp, respectively (Supplementary Text S3). XZ5 has seven minor-type root hair-specific *cis*-elements (RHEs) and three major-type RHEs in its promoter, one more minor-type RHE than Tadmor at a position of −1454 from the transcription start site of *HvEXPB7*, and two more RHEs (one minor-type at position −1454 and one major-type at position −468) compared with XZ54 (Supplementary Fig. S5). In addition, a comparison of the *HvEXPB7* DNA sequences from the start codon (ATG) to the stop codon (TGA) of XZ5, XZ54 and Tadmor (GenBank: KT000616, KT000617 and KT000618 will be released on 1 June 2016) showed 22 single nucleotide polymorphism sites (SNPs), producing 14 amino acid site divergences (Table 1, Supplementary Text S3), which might cause the differences in the protein structure, thereby affecting function. There are 21 SNPs (14 amino acid polymorphic sites) between XZ5 and Tadmor; however, there are only four SNPs (three amino acid polymorphic sites) between XZ54 and Tadmor, but 19 SNPs (11 amino acid polymorphic sites) were found between the two wild barley genotypes (Table 1, Supplementary Text S3).

**Table 1.** The amino acid polymorphism of HvEXPB7 for the three genotypes XZ5, XZ54 and Tadmor

**Genotype**	**Amino acids polymorphism**													
	4	9	10	12	24	47	57	58	60	66	69	73	81	261
XZ5	Ala	Ser	Val	Ala	Cys	Asp	Leu	Ala	Pro	Ser	Ala	Met	Ala	Asp
XZ54	Thr	Ala	Ile	Val	Ser	Asn	Pro	Ala	Thr	Ala	Thr	Thr	Ala	Asp
Tadmor	Thr	Ala	Ile	Val	Ser	Asn	Pro	Thr	Thr	Ala	Thr	Thr	Thr	Glu

### Tissue expression pattern and subcellular localization of *HvEXPB7*

Tissue-specific expression of the *HvEXPB7* gene of XZ5 was examined via RT-PCR in different organs, including root, stem, leaf and seed (Fig. 7B). The transcript of *HvEXPB7* was preferentially accumulated in the roots of XZ5, and only low levels of expression were detected in the other three tissues.

Using the programs SignalP 4.1 Server and WoLF-PSORT, it was predicted that HvEXPB7 had a 25bp signal peptide at the N terminal of the protein sequence for entry into the secretory pathway. To investigate the subcellular localization of HvEXPB7, the GFP reporter gene translationally fused to the HvEXPB7 coding region was used in a transient assay in tobacco leaf epidermis and onion epidermis cells. The fused green fluorescence completely overlapped the RFP red fluorescence of a marker protein, pm-rb CD3-1008 (Xu *et al.*, 2014), specifically located on the plasma membrane in tobacco leaf epidermis cells (Fig. 7C). After cell plasmolysis by addition of a 30% sucrose solution, a laser confocal scanning microscope was used to check whether HvEXPB7 was located in the cell wall or the plasma membrane in onion epidermis cells. The results clearly indicated that HvEXPB7 is located in the plasma membrane (Fig. 7D).

### Silencing *HvEXPB7* leads to significant repression of root hair growth

To obtain direct evidence for the function of *HvEXPB7*, BSMV-VIGS-based gene silencing technology was used to determine whether disruption of this gene at the mRNA level affects root hair growth in barley. First, the recombinant BSMV virus was constructed with the barley phytoene desaturase gene (*HvPDS*) and was tested on XZ5. In the BSMV:HvPDS-inoculated plants, the photo-bleaching symptoms were obviously exhibited by 21 d post-inoculation (dpi) (Supplementary Fig. S6A). In contrast, the leaves in the mock-inoculated barley plants developed normally during the observation period. Approximately 94.7% of the *HvPDS* transcripts were suppressed in the BSMV:HvPDS-inoculated plants compared to the mock-inoculated plants (Supplementary Fig. S6B). These results showed that the BSMV-VIGS system could be used to assess the potential effects of *HvEXPB7* after silencing its expression.

**Fig. 8.**
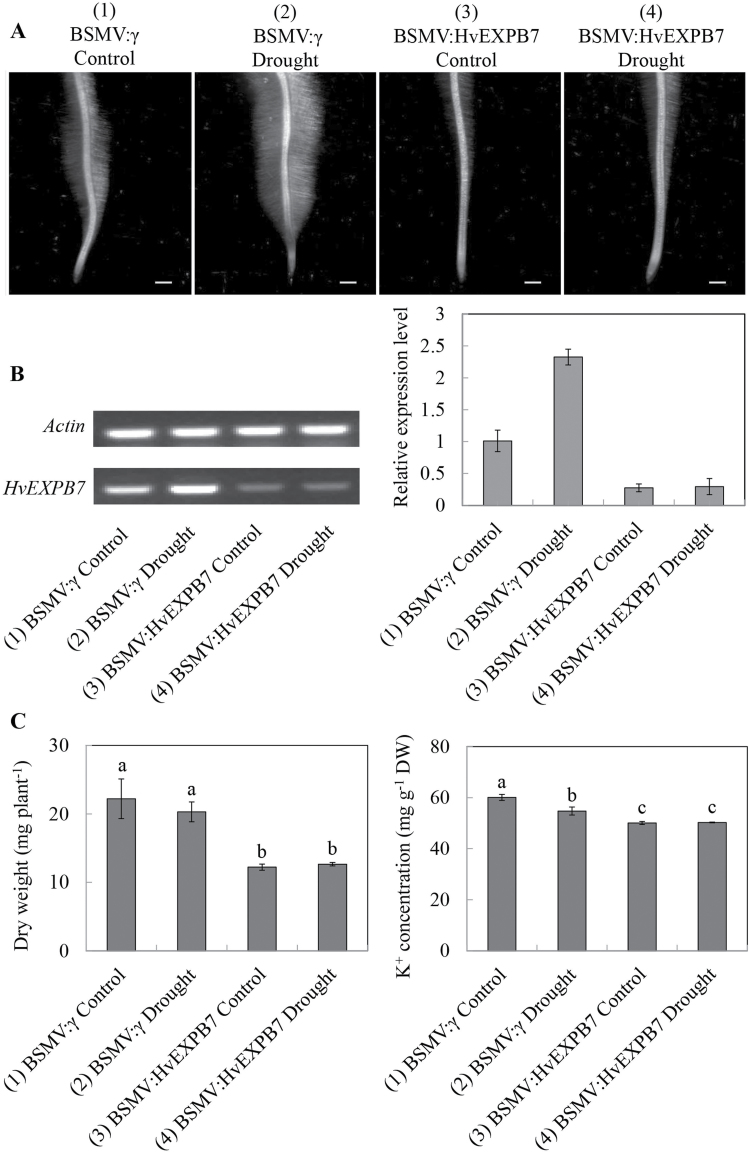
Functional assessment of *HvEXPB7* in wild barley XZ5 via BSMV-VIGS. (A) Root hair morphology observation through a stereomicroscope. (B) RT-PCR analysis of the relative transcript levels of *HvEXPB7* in XZ5 roots. (C) Dry weight and K^+^ concentration in roots of mock and BSMV:HvEXPB7-inoculated XZ5 seedlings. (1) Roots of BSMV:γ mock-inoculated seedlings grown in BNS for 21 d. (2) Roots of BSMV:γ mock-inoculated seedlings grown in BNS for 16 d, then treated by PEG 6000 for 5 d. (3) Roots of BSMV:HvEXPB7-inoculated seedlings grown in BNS for 21 d. (4) Roots of BSMV:HvEXPB7-inoculated seedlings grown in BNS for 16 d, then treated by PEG 6000 for 5 d. Scale bars, 1mm.

As shown in Fig. 8A, drought-stress induced root hairs in both the mock- and BSMV:HvEXPB7-inoculated XZ5 plants compared with their controls (Fig. 8A, treatment 2 vs. 1, and 4 vs. 3). The RT-PCR results showed that the expression levels of *HvEXPB7* in the mock- and BSMV:HvEXPB7-inoculated XZ5 plants under drought stress were 2.3 and 1.1 times higher than their controls (Fig. 8B, treatment 2 vs. 1, and 4 vs. 3). The transcription level of *HvEXPB7* in the roots was knocked down by 72.9% and 87.4% in the roots of the BSMV:HvEXPB7-inoculated seedlings compared to the roots of the mock-inoculated seedlings under the control and drought conditions, respectively (Fig. 8B, treatment 3 vs. 1, treatment 4 vs. 2). The stereomicroscopic images showed that the BSMV:HvEXPB7-inoculated seedlings under the control (treatment 3) and drought (treatment 4) conditions had very short and small root hairs compared to the mock-inoculated barley plants (treatment 1 and 2) at 21 dpi (Fig. 8A), when the visual symptoms of photo-bleaching were clearly noticed in the most BSMV:HvPDS-inoculated plants. In addition, compared to the roots of the mock-inoculated seedlings, dry weight and K^+^ concentration in roots of the BSMV:HvEXPB7-inoculated seedlings decreased by 45.0% and 16.6% under control, and by 37.8% and 8.2% under drought conditions (Fig. 8C, treatment 3 vs. 1, treatment 4 vs. 2). Obviously, the result proved that *HvEXPB7* is closely associated with barley root hair development, subsequently influencing root biomass and K^+^ absorption.

## Discussion

### Drought stress significantly improved root hair development in XZ5

Root hairs are slender projections originating from the epidermal cells, and they have important function in water/nutrient uptake (Brown *et al.*, 2012). Development of root hairs in plants is affected by environmental factors (Niu *et al.*, 2011; Delhaize *et al.*, 2012). Drought is one of the main abiotic stresses affecting plant growth and crop yield worldwide. Therefore, it is vital to find approaches to improve drought tolerance of crops. In this study, the stereomicroscope and SEM results (Fig. 1) showed that water stress caused by 20% PEG 6000 greatly induced root hair development in drought-tolerant Tibetan wild barley XZ5, but variously inhibited root hair development in XZ54 (drought sensitive) and cv. Tadmor (drought tolerant). Root scanning experiment showed that the sensitive genotype XZ54 recorded the longer root length and more surface area than XZ5 and Tadmor under drought and control conditions. In addition, 20% PEG induced drought had little effect on root length and surface area of the three genotypes (Supplementary Fig. S7). These results implied that the higher drought tolerance in XZ5 could be attributed to its well-developed root hairs, and XZ5 and Tadmor might differ in drought tolerance mechanisms, resulting in larger and more root hairs in XZ5.

### Comparative root hair transcriptome analysis reveals key genes associated with root hair growth in XZ5

Comparative transcriptome analysis of the genotypes differing in drought tolerance is a promising strategy to identify the transcripts responsible for the tolerance. Currently, the comparative root hair transcriptome profiling was firstly performed in XZ5, XZ54 and cv. Tadmor, and 36 putative drought-tolerance-associated genes were identified (Fig. 3). Based on the identified genes, an integrated schematic diagram of the potential mechanisms involved in drought tolerance in XZ5 was produced (Fig. 4). The genes most likely to be involved in drought stress will be further studied to elucidate the components of the drought-tolerant mechanism in barley.

#### Energy-related DEGs

ATP is the most important source of energy and involved in diverse physiological processes in plant growth and development, including vegetative growth (Wolf *et al.*, 2007), biotic/abiotic stress responses (Chivasa *et al.*, 2009) and cell viability (Chivasa *et al.*, 2005). It has been shown that extracellular ATP plays a role in the polarized growth of root hairs through increasing production of reactive oxygen species (ROS) at the tip of the *Medicago truncatula* hairs, and application of extracellular ATP effectively promoted root hair growth (Kim *et al.*, 2006). Five of the 36 root hair DEGs encoding the ATP synthase beta subunit and ATP sulfurylase were identified in this study. All of them were up-regulated in the two drought-tolerant genotypes but were unaltered in the drought-sensitive genotype under drought stress, indicating their important roles in enhancing drought tolerance and that they might have a certain effect on root hair development in XZ5.

#### Antioxidant- and stress-related DEGs

Several studies have indicated that a high antioxidative capacity is responsible for drought resistance in plants. The presence of ROS in the growing cells is an important signal, but at the same time, this results in the development of an antioxidant defence by antioxidase, like peroxidase, to prevent oxidative damage to the cells (Csiszar *et al.*, 2012). Won *et al.* (2009) proved that peroxidases can coordinate the production of hydroxyl radicals necessary for the initial cleavage of the cell wall at the site of root hair initiation in the trichoblast. The peroxidases identified in our study may be involved in the ROS signalling pathways, which in turn, impact root hair development and drought tolerance in XZ5. Expression of the *LEA* gene is usually associated with plant response to dehydration, although some LEA proteins may play a role as antioxidants. The silencing of *SAG21/AtLEA5*, one of the *LEA* gene family members, exhibits earlier flowering and senescence, decreases shoot biomass and primary root length and short root hair length in *Arabidopsis* (Salleh *et al.*, 2012). In this study, two *LEA* genes, expressing very differently between XZ5 and Tadmor in response to drought stress, were considered to be specific drought tolerance-related genes in XZ5, contributing to its drought tolerance and even to its well-developed root hairs.

#### Cell wall development-related DEGs

Xyloglucan endotransglucosylase hydrolases (XTHs) are enzymes involved in the modification of load-bearing cell wall components. Decrease in *AtXTH18* mRNA abundance by RNAi results in some reduction of the epidermal cell length in the primary root (Osato *et al.*, 2006). In addition, high expression of a *Brassica campestris* homologue of *AtXTH9* in *Arabidopsis* evokes a pronounced increase in cell expansion (Shin *et al.*, 2006), and loss of function of *AtXTH21* restricts the growth of primary roots and causes an obvious dwarf phenotype in *Arabidopsis* (Liu *et al.*, 2007). It is shown that *Arabidopsis GL2* is able to directly regulate *AtXTH17* expression through an L1-box sequence in its promoter of *gl2* mutants during root hair formation (Tominaga-Wada *et al.*, 2009). Three homologues of cell wall development associated genes, whose expression were significantly induced in XZ5 under drought stress compared with XZ54 and Tadmor, were identified in this study. So it is predicted that this type of gene may be involved in cell wall modification and formation, leading to root hair development in XZ5 and to better tolerance to drought stress.

### *HvEXPB7* cloned from XZ5 has abundant genetic diversity

Previously, it was found that proteins from the EXPA family could loosen eudicot cell walls but had less effect on grass cell walls, and grass pollen EXPB proteins showed the reverse pattern (Sampedro *et al.*, 2015). In the present study, a novel β-expansin gene, named *HvEXPB7*, was the unique root hair development related gene in the identified drought tolerance-associated genes, and was differently expressed between XZ5 and Tadmor under drought. Therefore, the β-expansin gene *HvEXPB7* from XZ5 was cloned and characterized (Fig. 5; Supplementary Fig. S4). *HvEXPB7* proved to be a real member of the β-expansin family as had conserved motifs and high homology with other EXPBs (Figs 6, 7A; Supplementary Texts S1, S2). Yu *et al.* (2011) predicted that the missense mutation in *OsexpA17*, resulting in a change of Gly (104) to Arg, occurs adjacent to Cys at position 103, causing a change in the amino acid sequence, leading to short root hair length. As shown in Table 1, there were 14 amino acid site variations among the three barley genotypes, and XZ5 reserved abundant genetic diversity compared to XZ54 and Tadmor. These differences may alter the protein structure, thereby affecting function. Promoter deletion analyses demonstrated that the RHE motifs are necessary for root hair specific expression of EXPB promoters, such as *OsEXPB5* and *HvEXPB1* (Won *et al.*, 2010), and the number of RHEs rather than their orientation has a great influence on gene function (Kim *et al.*, 2006). The comparison of promoters of XZ5, XZ54 and Tadmor showed different numbers of RHEs, and in particular, XZ5 had one more RHE motif than Tadmor and two more RHEs than XZ54 (Supplementary Fig. S5; Supplementary Text S3), which might enhance expression of *HvEXPB7* in XZ5 in response to drought stress. Although most of the tested expansin genes are located in the cell wall, *AtEXP7* and *OsEXPA17* were verified as located in the plasma membrane and having functions related to root hair growth (Yu *et al.*, 2011). This study found that the subcellular localization of *HvEXPB7* was in the plasma membrane (Fig. 7C, D). Therefore, it can be speculated that there may be some mechanisms allowing interaction between HvEXPB7 protein and cell wall components.

### *HvEXPB7* expression is associated with root hair growth

In recent years, reverse genetics technologies including VIGS have been widely employed to verify the function of specific genes (Lee *et al.*, 2012). The advantages of VIGS over traditional transgenic technologies make it a particularly useful tool for ‘loss-of-function’ gene analysis. BSMV-based vectors have proven to be effective for VIGS in monocots. BSMV was successfully used for gene silencing in wheat and barley (Scofield and Nelson, 2009). Here, a VIGS system based on BSMV vectors in wild barley accession XZ5 was developed. The control *HvPDS* gene silencing experiments showed a leaf photo-bleaching phenotype (Supplementary Fig. S6). Whether BSMV-mediated silencing of *HvEXPB7* could lead to inhibition of root hair growth constituting an opposite effect was also investigated. Indeed, *HvEXPB7* silencing produced a large decrease in root hair growth in XZ5 (Fig. 8). As shown in Fig. 8A and B, root hair growth in XZ5 was significantly enhanced under drought, while *HvEXPB7*-silenced plants developed very short and limited root hairs, even under drought stress. The result suggests that the root hair growth of XZ5 can be regulated by *HvEXPB7*. It is well-known that K^+^ is the second most abundant mineral nutrient in plants comprising 2−10% of the plant dry weight, and involved in numerous functions like drought tolerance and root hair elongation (Anschütz *et al.*, 2014; Daras *et al.*, 2015). Meanwhile, root hair plays an important role in drought tolerance, because of its important functions in water and K^+^ uptake. In order to investigate the gene silencing effect of *HvEXPB7*, the root dry weight and K^+^ concentration were measured. As shown in Fig. 8C, the root dry weight and K^+^ concentration of *HvEXPB7*-silenced plants were markedly decreased. These results suggest that up-regulation or over-expression of *HvEXPB7* enhances root hair growth thereby affecting K^+^ uptake, subsequently influencing the root biomass of XZ5 under drought stress.

In conclusion, the root hair morphology observation by stereomicroscope and SEM, the use of genome-wide transcriptome analysis on three barley genotypes with different drought-tolerant abilities combined with BSMV-VIGS function verification methods leads to the following conclusions: (i) XZ5 has significantly well-developed root hairs compared to XZ54 and cv. Tadmor under drought stress; (ii) XZ5 has far more DEGs than XZ54 and cv. Tadmor in response to drought; (iii) 36 drought tolerance-related DEGs are identified, and the full length sequence of a novel β-expansin gene named *HvEXPB7* is cloned; (iv) the *HvEXPB7* sequence from XZ5 has abundant genetic diversity compared to cv. Tadmor; (v) the *HvEXPB7* promoter of XZ5 has more RHEs than XZ54 and cv. Tadmor; (vi) BSMV-VIGS is successfully carried out on wild barley; (vii) *HvEXPB7* expression plays an important role in root hair development under drought stress. These results, to a degree, illustrate the drought tolerance mechanism of XZ5 and its differences from cv. Tadmor, which is useful for future crop genetic improvement. These results illustrate that *HvEXPB7* could be applied in biotechnology to improve drought tolerance via root hair growth in barley.

## Supplementary data

Supplementary data is available at *JXB* online.

Supplementary Fig. S1. The operation of root hair isolation.

Supplementary Fig. S2. Hierarchical cluster analysis of all 266 DEGs in root hairs of XZ5, XZ54 and Tadmor according to their relative expression levels in response to drought stress.

Supplementary Fig. S3. Expression profiles of five root hair genes by qRT-PCR in three barely genotypes.

Supplementary Fig. S4. The nucleotide and amino acid sequences of *HvEXPB7*.

Supplementary Fig. S5. Relative positions of RHEs in the *HvEXPB7* promoter regions of XZ5, XZ54 and Tadmor.

Supplementary Fig. S6. Silencing of the phytoene desaturase (*PDS*) gene in wild barley XZ5 using BSMV-VIGS.

Supplementary Fig. S7. Root scanning of the root length and root surface area of Tibetan wild barley XZ5, XZ54 and cv. Tadmor grown under control or drought conditions.

Supplementary Table S1. List of real-time PCR primers for RNA-Seq.

Supplementary Table S2. List of PCR primers for *HvEXPB7* cloning and function verification.

Supplementary Table S3. Summary of root hair read numbers of XZ5, XZ54 and Tadmor, and their results mapped to the barley genome.

Supplementary Table S4. DEGs up-regulated in XZ5 but down-regulated or unchanged in XZ54, or no change in XZ5 but down-regulated in XZ54.

Supplementary Table S5. DEGs up-regulated in XZ54 but down-regulated or unchanged in XZ5, or unchanged in XZ54 but down-regulated in XZ5.

Supplementary Table S6. DEG up-regulated in both XZ5 and XZ54.

Supplementary Table S7. DEGs down-regulated in both XZ5 and XZ54.

Supplementary Table S8. DEGs only up- or down-regulated in Tadmor.

Supplementary Table S9. KEGG pathways of tolerance related root hair genes in response to drought stress.

Supplementary Text S1. Protein sequences used for alignment analysis in Fig. 6.

Supplementary Text S2. Protein sequences used for phylogenetic tree analysis in Fig. 7A.

Supplementary Text S3. Promoter and gene sequence of *HvEXPB7* in XZ5, XZ54 and Tadmor.

Supplementary Data
