# Supplementary material for: HvEXPB7, a novel β-expansin gene revealed by the root hair transcriptome of Tibetan wild barley, improves root hair growth under drought stress
Source: J Exp Bot. 2015 Sep 28;66(22):7405–19. doi: 10.1093/jxb/erv436 (PMC4765802; doi:10.1093/jxb/erv436)
Supplement: Supplementary Data [file supp_66_22_7405__index.html]

 HvEXPB7, a novel β-expansin gene revealed by the root hair transcriptome of Tibetan wild barley, improves root hair growth under drought stress — HvEXPB7, a novel β-expansin gene revealed by the root hair transcriptome of Tibetan wild barley, improves root hair growth under drought stress — Supplementary Data 

# *HvEXPB7*, a novel β-expansin gene revealed by the root hair transcriptome of Tibetan wild barley, improves root hair growth under drought stress

## Supplementary Data

Data files

- Supplementary Data - Supplementary Data
