# Supplementary material for: HvEXPB7, a novel β-expansin gene revealed by the root hair transcriptome of Tibetan wild barley, improves root hair growth under drought stress
Source: J Exp Bot. 2015 Sep 28;66(22):7405–19. doi: 10.1093/jxb/erv436 (PMC4765802; doi:10.1093/jxb/erv436)
Supplement: Supplementary Data [file supp_erv436_jexbot158196_file001.pdf]

HvEXPB7, a Novel β-Expansin Gene Revealed by Root Hair Transcriptome of Tibetan Wild Barley Improves Root Hair Growth under Drought Stress

Xiaoyan He, Jianbin Zeng, Fangbin Cao, Imrul Mosaddek Ahmed, Guoping Zhang, Eva Vincze, Feibo Wu

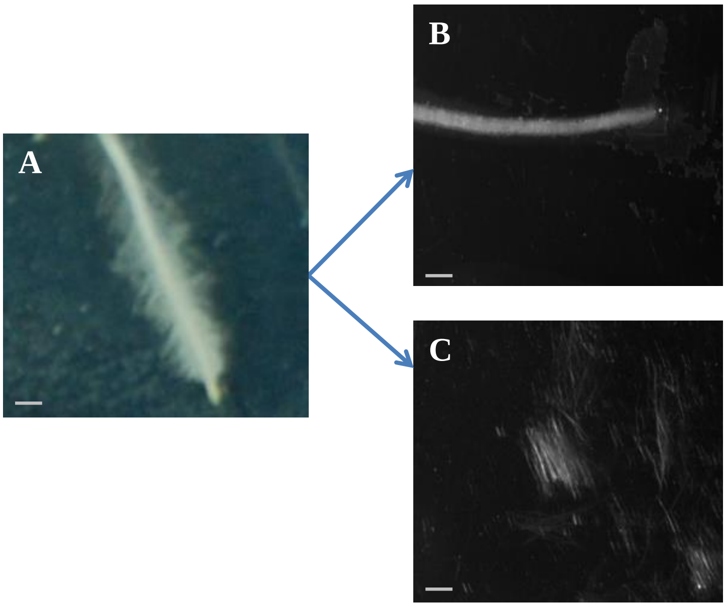

Fig. S1. Root hair isolation. (a) Root. (b) Stripped root. (c) Isolated root hair. Scale bars=1 mm.

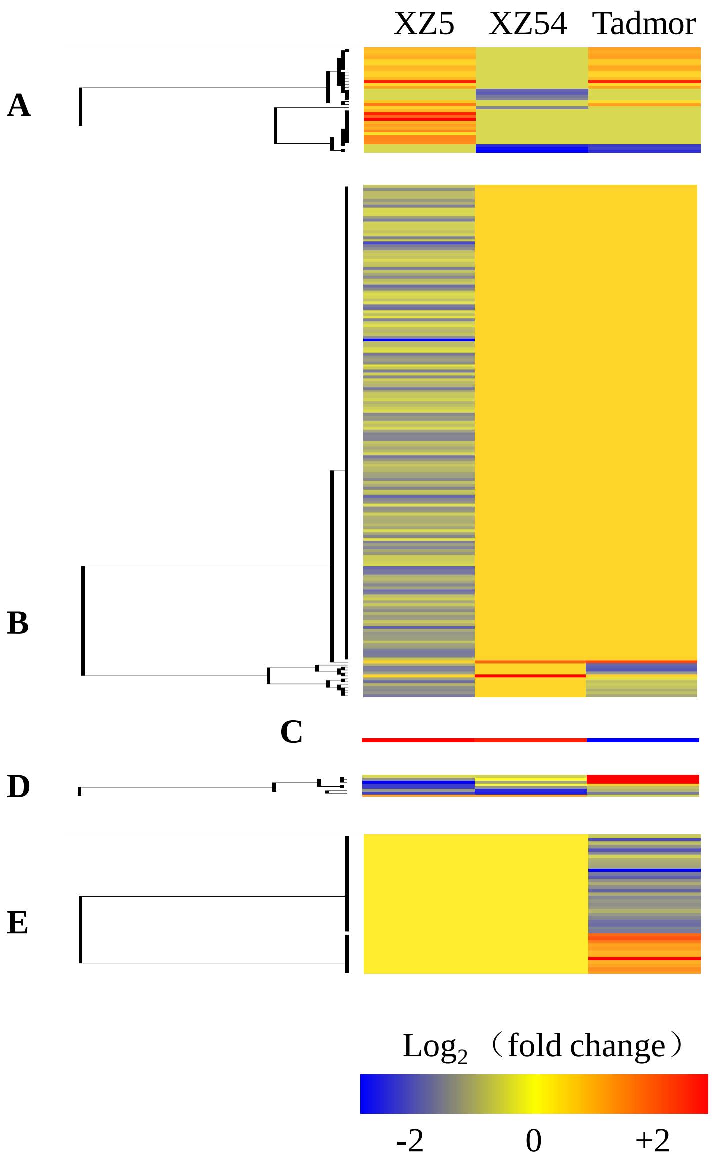

Fig. S2. Hierarchical cluster analysis of all 266 DEGs of root hairs of XZ5, XZ54 and Tadmor according to their relative expression levels in response to drought stress. (A) 36 DEGs up-regulated in XZ5 but down-regulated or unchanged in XZ54, or no change in XZ5 but down-regulated in XZ54. (B) 180 DEGs up-regulated in XZ54 but down-regulated or unchanged in XZ5, or unchanged in XZ54 but down-regulated in XZ5. (C) 1 DEG up-regulated in both of XZ5 and XZ54. (D) 8 DEGs down-regulated in both of XZ5 and XZ54. (E) 41 DEGs only up- or down-regulated in Tadmor. The relative expression levels and functions of these DEGs were listed in Supplementary Table S4-S8. Hierarchical clustering of DEGs was displayed by pearson correlation and pairwise average-linkage as a measurement of similarity.

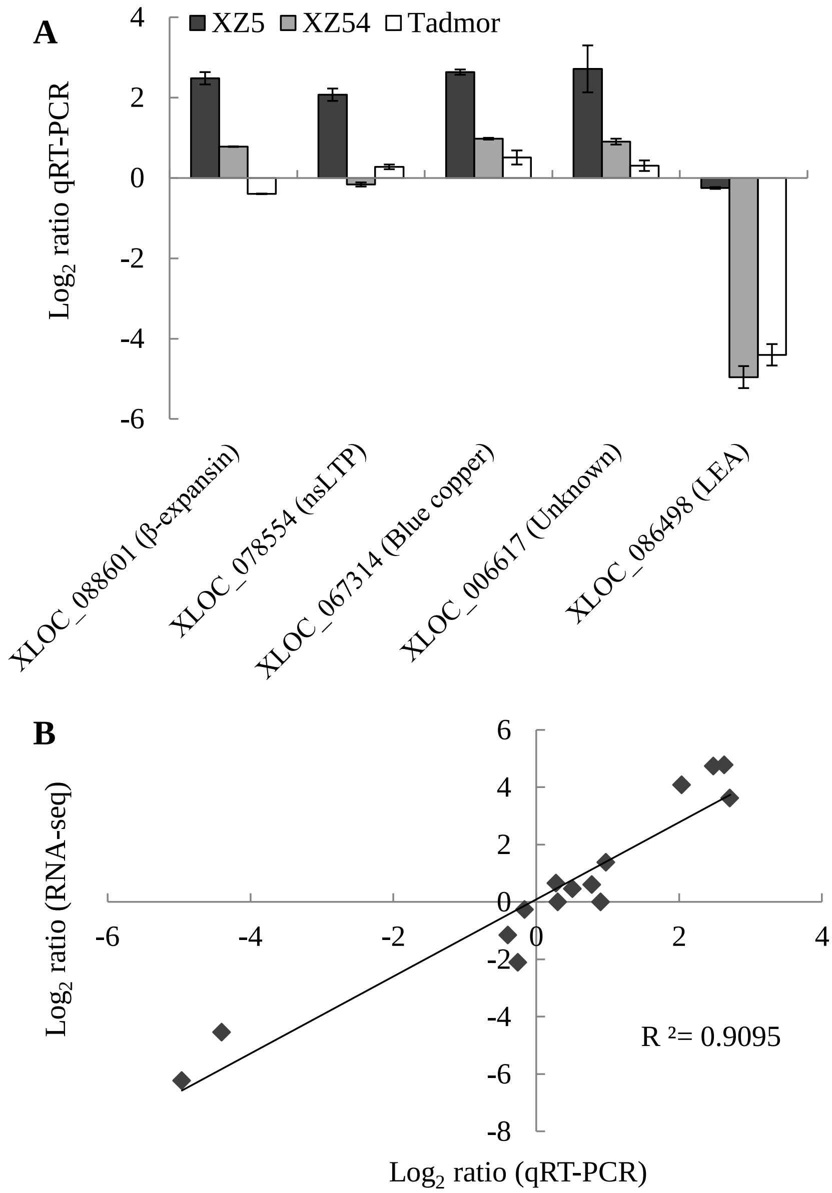

Fig. S3. Expression profiles confirmation of five root hair genes by qRT-PCR in three barely genotypes. (A) qRT-PCR of five genes in XZ5, XZ54 and Tadmor. The fold changes in gene expression were transformed to $\log_2$ scale (with three biological replications and three technology replications). (B) The qRT-PCR data log2-values (X-axis) were plotted against the RNA-seq log2 values (Y-axis) (R^2= 0.9095; $P < 0.001$).

```
1       aatctccccaacggcattaaccaccagagtagggtcatcgatcagtaaccaacgagaccagcaagcgagccatggcagcggccttttcct
                                                                                  M  A  A  A  F  S  S
91      cctactccgtcgcggccgctttcctctgcctgctcgccgccaacggctgctgcggctgcccctggtttctccctgctacattctgccctg
          Y  S  V  A  A  A  F  L  C  L  L  A  A  N  G  C  C  G  C  P  W  F  L  P  A  T  F  C  P  E
181     aaccgacacccgaccccatacctacacctgaccccgctccagctacacctccgcctgctctggctacccccagcccaggctccggctctg
          P  T  P  D  P  I  P  T  P  D  P  A  P  A  T  P  P  P  A  L  A  T  P  S  P  G  S  G  S  G
271     gcagcgccaacggttccatgggcggctggctggacgccagggccacatggtacggcgctccggatggcgccgggccgttggacaacggtg
          S  A  N  G  S  M  G  G  W  L  D  A  R  A  T  W  Y  G  A  P  D  G  A  G  P  L  D  N  G  G
361     gcgcgtgcgggttcaagaacgtgaacctgccgcccttcaacgccatgacgtcgtgcggcaacgagccgatcttcaaggacggcaagggat
          A  C  G  F  K  N  V  N  L  P  P  F  N  A  M  T  S  C  G  N  E  P  I  F  K  D  G  K  G  C
451     gcggctcctgctaccagataaggtgcgtgggcaaggttcacccagcgtgctccggcgaccccgagacggtgatcatcaccgacatgaact
          G  S  C  Y  Q  I  R  C  V  G  K  V  H  P  A  C  S  G  D  P  E  T  V  I  I  T  D  M  N  Y
541     actacccggtcgcccgctaccacttcgacctcagcggcactgccttcggcgccatggccaaggacggccgcaacgacgagctccgccacg
          Y  P  V  A  R  Y  H  F  D  L  S  G  T  A  F  G  A  M  A  K  D  G  R  N  D  E  L  R  H  A
631     ccggcatcatcgacatgcagttcaagagggtgccgtgccagtacacagggctgtccgtgacgtttcacgtggagaaggggtcgaacccga
          G  I  I  D  M  Q  F  K  R  V  P  C  Q  Y  T  G  L  S  V  T  F  H  V  E  K  G  S  N  P  N
721     attacctggcgatcctggtggagtacgggaacggcgacggcgacgtggcgcaggtggacctcatggacggcggcgagccaacggtgtcct
          Y  L  A  I  L  V  E  Y  G  N  G  D  G  D  V  A  Q  V  D  L  M  D  G  G  E  P  T  V  S  W
811     ggaggccgatgagggagtcctggggctccatctggcgcctggacacgcggcgcccgcttcgggggcccttctcgctgcgggtcaccaacg
          R  P  M  R  E  S  W  G  S  I  W  R  L  D  T  R  R  P  L  R  G  P  F  S  L  R  V  T  N  G
901     ggtccggcaggtcgctcgtcgcggaccaggtcatccccgccgactggcagcccgacaccgtgtacagctccgacgtccagttcgatgaat
          S  G  R  S  L  V  A  D  Q  V  I  P  A  D  W  Q  P  D  T  V  Y  S  S  D  V  Q  F  D  E  *
991     gatcccgtccgatgatccatggaaggcatgcacgtacgtccatgcctgcatggatttttttgtgccaagctatatagctgagacgtcgcct
1081    gtgacttccggtgcaagtcctcgtcgtgtgctacctatgctacatatgtacacgtagtacataacatacgtggcagctttaattagtttg
1171    tgggatctggagtaggcagcgagtagtatatattgctcgcagctataaatttacgtttgtaatttctcctgtattacacaagtttattat
1261    gtcactggtgtgtataaa
```

Fig. S4. The nucleotide and amino acid sequences of *HvEXPB7*.

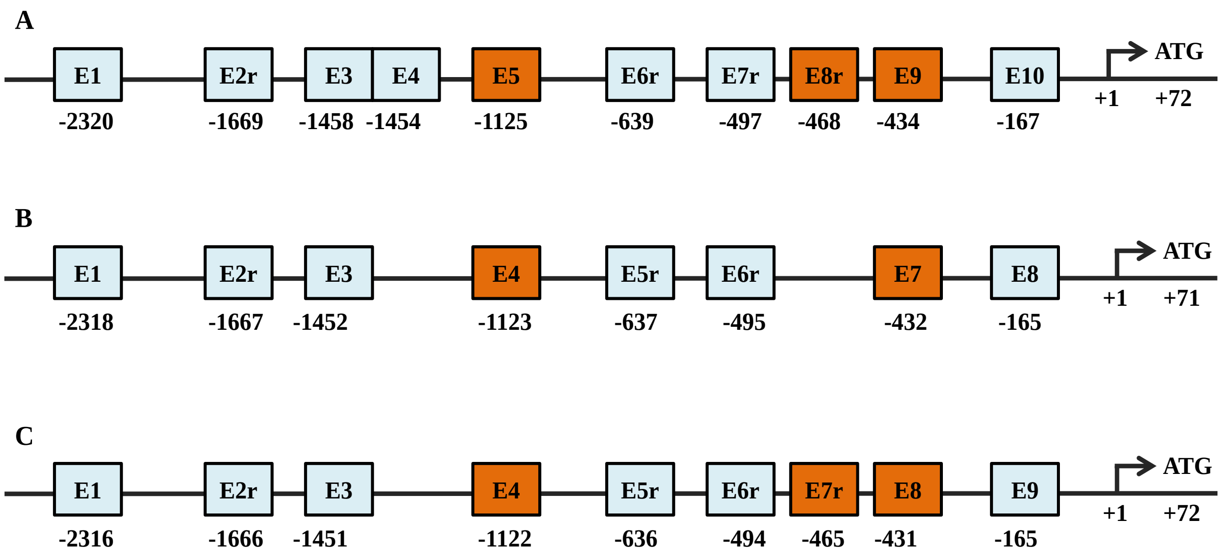

Fig. S5. Relative positions of RHEs in the *HvEXPB7* promoter regions of XZ5, XZ54 and Tadmor. (A) XZ5. (B) XZ54. (C) Tadmor. Numbers below the sequence represent the nucleotide positions of the RHE motifs (light blue and orange red boxes) relative to the putative transcription initiation site (+1). 'r' indicates that the RHE is in the reverse direction. RHEs with major- and minor-type consensus sequences are boxed in orange red and light blue, respectively.

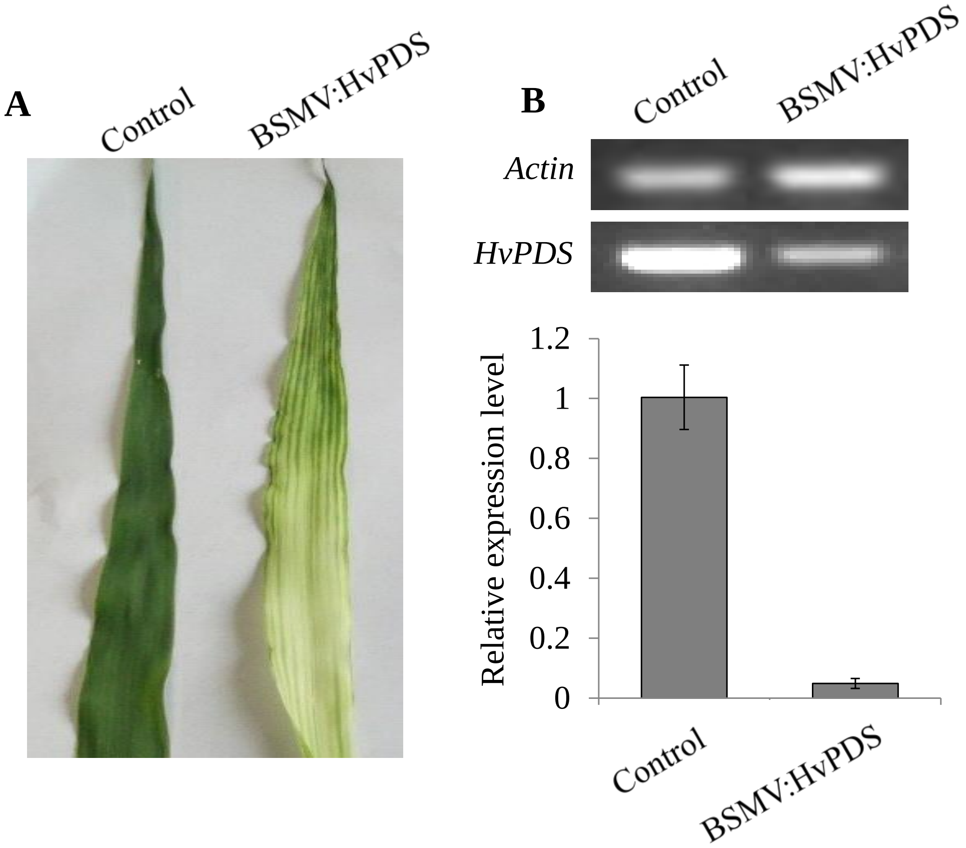

Fig. S6. Silencing of the phytoene desaturase (*PDS*) gene in wild barley XZ5 using BSMV-VIGS. (A) Photobleaching symptom of BSMV:HvPDS inoculated plants in contrast to those treated with empty vectors. Photograph was taken 21 dpi. (B) RT-PCR analysis of *HvPDS* transcript abundance in leaves of XZ5 21 dpi.

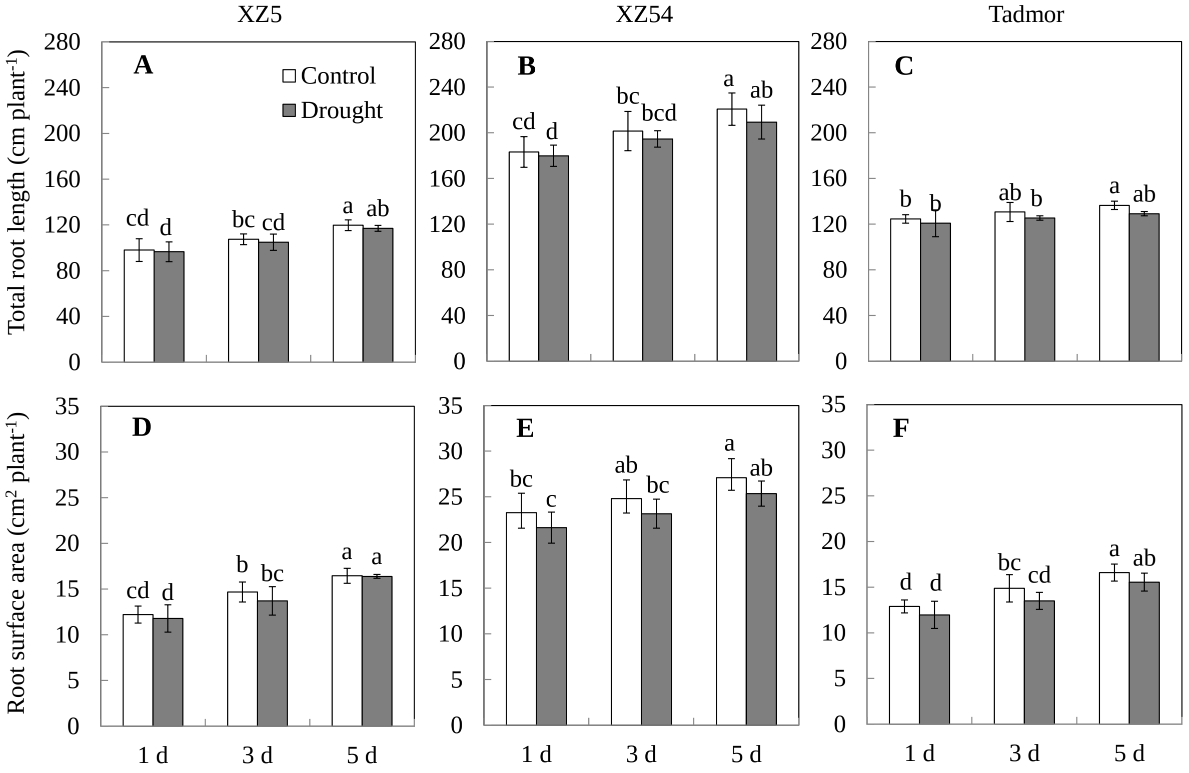

Fig. S7. Root scanning of the root length (A, B, C) and root surface area (D, E, F) of Tibetan wild barley XZ5 (left panel), XZ54 (middle panel) and *cv.* Tadmor (right panel) grown under control or drought conditions. Error bars represent SD values (n=6). Different letters indicate significant differences (P<0.05) among the treatments within each genotype.

Table S1 List of real-time PCR primers for confirmation of RNA-Seq

**Gene Id**	**Description**	**Forward primer（5′ → 3′）**
XLOC_088601	Beta-expansin 1a expressed	Forward primer: GCCTTGATGCCAATCACCAG
		Reverse primer: TGGTTAATGCCGTTGGGGAG
XLOC_078554	Non-specific lipid-transfer protein	Forward primer: ATCTCACCCCCTAACACCCA
	precursor	Reverse primer: ATGGCGTCTCTGGAACTCTG
XLOC_067314	Blue copper	Forward primer: ACGCGGAATCCAGTTACTAGC
		Reverse primer: CACAAGCAGCGTGATCAGAG
XLOC_006617	Unknown	Forward primer: GCACCGCCAGGAAGAAAAAG
		Reverse primer: ATGTAGCGCACAAGGAACGA
XLOC_086498	Similarity to late embryogenesis	Forward primer: ACCCATCCTTCTCCACCAGT
	abundant protein (LEA)	Reverse primer: CGTACGGCCAGTACAACACT
Actin		Forward primer: TGGCTGACGGTGAGGACA
		Reverse primer: CGAGGGCGACCAACTATG

Table S2 List of PCR primers for HvEXPB7 cloning and function verification

**Primer name**	**Primer sequence (5' → 3')**
GSPF	TTCAAGGACGGCAAGGGATG
GSPR	GTTCGACCCCTTCTCCACGT
UPM-S	CTAATACGACTCACTATAGGGC
UPM -L	CTAATACGACTCACTATAGGGCAAGCAGTGGTATCAACGCAGAGT
EXPB7F	CAATCTCCCCAACGGCATTAAC
EXPB7R	TTTATACACACCAGTGACATAAT
EXPB7-promoter-F	TTCGCAAGTTTAAATCATCACAAG
EXPB7-promoter R	CAACGAGACCAGCAAGCGAGCC
EXPB7-CDS-F	ATGGCAGCGGCCTTTTCCTC
EXPB7-CDS-R	TCATTCATCGAACTGGACGT
EXPB7-35S:sGFP-F	CTCAGGTACCATGGCAGCGGCCTTTTCC
EXPB7-35S:sGFP-R	CTAGTCTAGATTCATCGAACTGGACGTC
EXPB7-γ-F	GTACGCTAGCACCCCATACCTACACCTG
EXPB7-γ-R	GTACGCTAGCATCCCTTGCCGTCCTTG
PDS-γ-F	GTACGCTAGCCGACGAGGTTTTTATTGC
PDS-γ-R	GTACGCTAGCAGTTATTTGAGTCCCGTC
EXPB7-RT-PCR-F	TCAAGAACGTGAACCTGCCG
EXPB7-RT-PCR-R	CTTGCCCACGCACCTTATCT
PDS- RT-PCR -F	AGTCTTTGGGTGGTGAGGTC
PDS- RT-PCR -R	CTTGAAGATATCGACTGGTG
γ-stain-F	CAACTGCCAATCGTGAGTAGG
ActinF	TGGCTGACGGTGAGGACA
ActinR	CGAGGGCGACCAACTATG

Table S3 Summary of root hair reads numbers of XZ5, XZ54 and Tadmor, and their mapped results to barely genome

Sample	Genotype					
	XZ5		XZ54		Tadmor	
	Control	Drought	Control	Drought	Control	Drought
Raw reads	29476018	26423412	27690596	27698298	29397348	24862762
Clean reads	29433186	26415212	27682280	27689364	29385518	24854408
Clean data rate	96.00%	97.43%	97.42%	97.38%	97.30%	97.32%
Total mapped reads	29433186	26415212	27682280	27689364	29385518	24854408
Uniquely mapped	20547478	15475014	19158705	21098069	21871926	14753813
Multiple mapped reads	3832360	7075850	4007429	3256107	3656852	6376676
Unmapped reads	5048859	3862596	4515124	3333126	3855525	3722213
Exp_gene_number	46383	43005	45318	45145	45247	42570

Table S4 DEGs up-regulated in XZ5 but down-regulated or unchanged in XZ54, or no change in XZ5 but down-regulated in XZ54

GeneId	Log_2(fold change)			Description
	XZ5	XZ54	Tadmor	
**Energy**				
XLOC_004739	3.24	—	3.95	ATP synthase subunit beta
XLOC_006853	2.58	—	2.48	ATP synthase subunit beta
XLOC_012447	3.66	—	3.99	ATP synthase subunit beta
XLOC_013896	3.69	—	3.68	ATP sulfurylase
XLOC_016879	3.33	—	3.92	ATP synthase subunit beta
XLOC_013018	3.40	—	3.24	Plastid glutamine synthetase 2
**Cell wall modification and root hair development**				
XLOC_024622	3.35	—	—	Cell wall-associated hydrolase
XLOC_031639	5.79	—	—	Xyloglucan endotransglycosylase hydrolase protein 8
XLOC_012543	2.42	—	2.75	Cell wall-associated hydrolase
XLOC_088601	4.74	—	—	Beta-expansin 1a expressed
**Transcription**				
XLOC_004913	3.00	—	3.65	rRNA intron-encoded homing endonuclease
XLOC_006324	2.50	—	2.39	rRNA intron-encoded homing endonuclease
**Metabolism**				
XLOC_094366	2.06	—	—	Serine acetyltransferase
XLOC_012656	2.50	—	2.72	Aldolase-type TIM barrel family protein isoform 1
XLOC_018005	2.75	—	2.29	Leucine rich protein
XLOC_020158	8.83	—	—	Small glutamine-rich tetratricopeptide repeat-containing protein beta
**Stress defense**				
XLOC_014532	7.86	—	7.60	Cytochrome p450 liketbp
XLOC_019100	4.86	—	—	Peroxidase 52
XLOC_032107	3.32	—	—	Cold-regulated protein blt14
XLOC_085743	—	-3.61	—	Peroxidase 12
XLOC_086498	—	-6.23	-4.55	Similarity to late embryogenesis abundant protein
XLOC_088358	—	-5.31	-4.90	Similarity to late embryogenesis abundant protein
**Unknown**				
XLOC_040065	2.58	-2.59	—	Conserved domain partial
XLOC_049979	2.31	—	2.18	Conserved protein
XLOC_057865	—	-3.00	—	ORF16-lacz fusion protein
XLOC_062616	3.36	—	3.82	Hypothetical protein
XLOC_088641	—	-6.68	-5.28	Predicted protein
XLOC_003324	—	-3.80	—	Predicted protein
XLOC_006617	3.63	—	—	---NA---
XLOC_064342	—	-2.55	—	Conserved protein
XLOC_060028	5.14	—	4.03	Conserved hypothetical protein
XLOC_028401	4.57	—	—	Hypothetical protein ZEAMMB73_284819
XLOC_025502	3.29	—	3.68	ORF16-lacz fusion protein

— represents no change.

Table S5 DEGs up-regulated in XZ54 but down-regulated/unchanged in XZ5, or unchanged in XZ54 but down-regulated in XZ5

Gene Id	Log$_2$(fold change）			Description
	XZ5	XZ54	Tadmor	
**Protein synthesis**				
XLOC_075318	-2.74	—	—	40s ribosomal protein s10
XLOC_079203	-3.26	—	—	40s ribosomal protein s11
XLOC_020875	-2.47	—	—	40s ribosomal protein s12
XLOC_070718	-3.36	—	—	40s ribosomal protein s13
XLOC_039539	-2.70	—	—	40s ribosomal protein s14
XLOC_046148	-3.90	—	—	40s ribosomal protein s14
XLOC_081223	-3.64	—	—	40s ribosomal protein s15
XLOC_054162	-3.55	—	—	40s ribosomal protein s15a-1-like
XLOC_082918	-3.75	—	—	40s ribosomal protein s18
XLOC_032123	-2.85	—	—	40s ribosomal protein s19
XLOC_040402	-3.23	—	—	40s ribosomal protein s23
XLOC_092443	-3.71	—	—	40s ribosomal protein s23
XLOC_007060	-3.32	—	—	40s ribosomal protein s24
XLOC_054218	-3.73	—	—	40s ribosomal protein s24
XLOC_015079	-3.39	—	—	40s ribosomal protein s25-1
XLOC_083823	-2.34	—	—	40s ribosomal protein s26
XLOC_052949	-3.47	—	—	40s ribosomal protein s27
XLOC_000608	-3.77	—	—	40s ribosomal protein s27a
XLOC_069430	-3.56	—	—	40s ribosomal protein s28
XLOC_013012	-3.64	—	—	40s ribosomal protein s30
XLOC_008838	-7.72	—	—	40s ribosomal protein s3a
XLOC_073619	-3.86	—	—	40s ribosomal protein s3a-like
XLOC_048775	-3.87	—	—	40s ribosomal protein s4
XLOC_000670	-3.27	—	—	40s ribosomal protein s5
XLOC_094261	-2.91	—	—	40s ribosomal protein s6
XLOC_070590	-3.81	—	—	40s ribosomal protein s6
XLOC_077626	-2.74	—	—	40s ribosomal protein s9
XLOC_069897	-3.28	—	—	40s ribosomal protein sa
XLOC_086164	-4.07	—	—	60s acidic ribosomal protein p2a
XLOC_074236	-2.55	—	—	60s acidic ribosomal protein p3
XLOC_050223	-4.04	—	—	60s ribosomal protein l10a-1
XLOC_089139	-5.33	—	—	60s ribosomal protein l10a-1
XLOC_052453	-3.18	—	—	60s ribosomal protein l11-1
XLOC_085384	-2.72	—	—	60s ribosomal protein l12
XLOC_024978	-3.21	—	—	60s ribosomal protein l12
XLOC_000238	-3.97	—	—	60s ribosomal protein l13-2
XLOC_072997	-3.00	—	—	60s ribosomal protein l13a
XLOC_074061	-3.24	—	—	60s ribosomal protein l13a
XLOC_059199	-4.13	—	—	60s ribosomal protein l14-1
XLOC_070829	-3.95	—	—	60s ribosomal protein l15
XLOC_087363	-3.05	—	—	60s ribosomal protein l17
XLOC_027406	-2.97	—	—	60s ribosomal protein l18
XLOC_069091	-3.72	—	—	60s ribosomal protein l18a

Gene Id	Log$_2$(fold change)			Description
	XZ5	XZ54	Tadmor	
XLOC_068657	-3.40	—	—	60s ribosomal protein l21
XLOC_081378	-3.60	—	—	60s ribosomal protein l22-2
XLOC_052029	-2.50	—	—	60s ribosomal protein l23
XLOC_073457	-4.11	—	—	60s ribosomal protein l23
XLOC_087786	-2.90	—	—	60s ribosomal protein l24
XLOC_088045	-4.68	—	-2.44	60s ribosomal protein l27
XLOC_073413	-3.29	—	—	60s ribosomal protein l28
XLOC_044358	-3.48	—	—	60s ribosomal protein l28
XLOC_054228	-2.78	—	—	60s ribosomal protein l29
XLOC_079130	-2.73	—	—	60s ribosomal protein l31
XLOC_030675	-3.17	—	—	60s ribosomal protein l32-1
XLOC_023616	-2.59	—	—	60s ribosomal protein l34
XLOC_022235	-2.90	—	—	60s ribosomal protein l35
XLOC_086081	-3.08	—	—	60s ribosomal protein l36
XLOC_048731	-3.74	—	—	60s ribosomal protein l37
XLOC_071115	-3.72	—	—	60s ribosomal protein l37a
XLOC_076961	-4.29	—	—	60s ribosomal protein l37a
XLOC_000488	-3.29	—	—	60s ribosomal protein l39
XLOC_005927	-3.37	—	—	60s ribosomal protein l39
XLOC_064561	-2.74	—	—	60s ribosomal protein l4
XLOC_050541	-3.67	—	—	60s ribosomal protein l4
XLOC_071105	-4.67	—	—	60s ribosomal protein l44
XLOC_018594	-4.97	—	—	60s ribosomal protein l44
XLOC_052973	-3.37	—	—	60s ribosomal protein l5
XLOC_077183	-2.90	—	—	60s ribosomal protein l6
XLOC_034007	-3.72	—	—	60s ribosomal protein l7-1
XLOC_038379	-3.43	—	—	60s ribosomal protein l7-2
XLOC_048797	-3.44	—	—	60s ribosomal protein l7a
XLOC_072252	-3.38	—	—	60s ribosomal protein l7a-like
XLOC_077210	-2.83	—	—	60s ribosomal protein l8
XLOC_039836	-3.88	—	—	60s ribosomal protein l9
XLOC_070264	-3.09	—	—	ribosomal protein l3
XLOC_053311	-3.88	—	—	ribosomal protein l3
XLOC_014690	-5.68	—	—	ribosomal protein l35a
XLOC_077690	-2.62	—	—	ribosomal protein s8e family protein
XLOC_089142	-3.53	—	—	ubiquitin-60s ribosomal protein l40-like
XLOC_067175	-3.57	—	—	h aca ribonucleoprotein complex subunit 1-like protein 1
**Metabolism**				
XLOC_067835	-4.15	—	-6.62	n- acetylneuraminyllactose-wheat germ agglutinin isolectin complexes
XLOC_023275	-4.81	—	-5.02	bowman-birk type trypsin inhibitor
XLOC_084419	-4.85	—	—	btb poz domain-containing protein npy2-like
XLOC_082237	-4.45	—	—	enoyl
XLOC_059664	-5.82	—	—	floral organ regulator partial
XLOC_072581	-4.00	—	-3.28	fructan:fructan 1-fructosyltransferase
XLOC_066763	-5.69	—	—	glucan endo- -beta-glucosidase-like protein 3-like
XLOC_066593	-4.71	—	—	glycine-rich rna-binding protein 2

Gene Id	Log_2(fold change）			Description
	XZ5	XZ54	Tadmor	
XLOC_084315	-3.75	—	—	pescadillo homolog
XLOC_088471	-3.14	—	—	plasminogen activator inhibitor 1 rna-binding protein
XLOC_020421	-5.96	—	-6.91	polygalacturonase precursor
XLOC_049760	-3.78	—	—	nucleolar protein 56-like
XLOC_058943	-2.71	—	—	nucleoside diphosphate kinase 1
XLOC_023030	-5.52	—	—	nucleotide-diphospho-sugar transferase
XLOC_059652	-3.52	—	—	probable nucleolar protein 5-2-like
XLOC_037868	-6.12	—	—	probable xyloglucan glycosyltransferase 3-like
XLOC_019557	-5.78	—	—	proliferating cell nuclear antigen
XLOC_080710	-5.03	—	—	xylanase inhibitor
XLOC_035315	-5.91	—	—	xyloglucan endotransglycosylase
XLOC_021898	-4.94	—	—	rrna 2 -o-methyltransferase fibrillarin 2-like
XLOC_032762	-10.20	—	—	soluble starch synthase ii-1
XLOC_072428	-3.29	—	—	sucrose:sucrose 1-fructosyltransferase
**Transcription**				
XLOC_088407	-4.42	—	—	iq calmodulin-binding motif family protein
XLOC_077254	-5.07	—	—	transcription factor
XLOC_002612	-4.90	—	—	histone
XLOC_030128	-5.07	—	—	histone
XLOC_004416	-5.10	—	—	histone
XLOC_004357	-6.06	—	—	histone
XLOC_091211	-6.49	—	—	histone
XLOC_056012	-4.81	—	—	histone h2a
XLOC_035520	-5.67	—	—	histone h2a
XLOC_002279	-4.67	—	—	histone
XLOC_050837	-6.96	—	—	histone h2ax
XLOC_090463	-3.87	—	—	histone h2b
XLOC_091768	-5.65	—	—	histone h2b
XLOC_000527	-4.46	—	—	histone h3
XLOC_079769	-4.49	—	—	histone h3
XLOC_004411	-4.84	—	—	histone h3
XLOC_004571	-5.24	—	—	histone h3
XLOC_037264	-5.51	—	—	histone h3
XLOC_044200	-5.93	—	—	histone h3
XLOC_031968	-6.72	—	—	histone h3
XLOC_017764	-5.48	—	—	histone H3.2
XLOC_000112	-4.30	—	—	histone h4
XLOC_002050	-4.33	—	—	histone h4
XLOC_020172	-6.01	—	—	histone h4
XLOC_053550	-6.64	—	—	histone partial
XLOC_027882	-5.73	—	—	histone superfamily protein
XLOC_078906	-3.99	—	—	probable histone h2a variant 3-like
**Cell growth**				
XLOC_079294	-5.51	—	-4.23	alpha-expansin 11 precursor family protein
XLOC_023702	-4.68	—	—	expansin a14
XLOC_081596	-2.73	—	—	elongation factor 1-beta

Gene Id	Log$_2$(fold change)			Description
	XZ5	XZ54	Tadmor	
XLOC_091348	-5.93	—	—	high mobility group b protein 7-like
XLOC_001013	—	7.54	—	cuticle protein
XLOC_007466	—	3.93	5.02	cortical cell-delineating protein
XLOC_058291	-5.27	—	-3.53	cortical cell-delineating protein precursor
XLOC_073825	-4.86	—	—	tubulin beta-1 chain-like
**Stress defense**				
XLOC_051168	-5.14	—	-3.68	pathogenesis-related protein pr-1-like
XLOC_069149	-4.35	—	—	peroxidase 12
XLOC_074244	-4.14	—	—	peroxidase 12-like
**Unknown**				
XLOC_052824	-5.11	—	—	hypothetical protein F775_23023
XLOC_086268	-3.86	—	—	hypothetical protein TRIUR3_19916
XLOC_055368	-3.11	—	—	---NA---
XLOC_030033	-4.39	—	—	---NA---
XLOC_066162	-4.59	—	—	---NA---
XLOC_062550	-4.59	—	—	---NA---
XLOC_052773	-4.64	—	—	---NA---
XLOC_063492	-4.64	—	—	---NA---
XLOC_018595	-4.77	—	—	---NA---
XLOC_063388	-5.07	—	—	---NA---
XLOC_020560	-5.33	—	—	---NA---
XLOC_042487	-5.42	—	—	---NA---
XLOC_036694	-5.42	—	—	---NA---
XLOC_086267	-5.48	—	—	---NA---
XLOC_024947	-5.60	—	—	---NA---
XLOC_029127	-5.66	—	—	---NA---
XLOC_035662	-5.70	—	—	---NA---
XLOC_041302	-5.71	—	—	---NA---
XLOC_086452	-5.79	—	-7.15	---NA---
XLOC_070581	-5.79	—	—	---NA---
XLOC_050650	-5.89	—	—	---NA---
XLOC_057217	-6.03	—	—	---NA---
XLOC_036913	-6.09	—	—	---NA---
XLOC_038742	-6.19	—	—	---NA---
XLOC_066253	-6.37	—	—	---NA---
XLOC_037637	-6.44	—	-3.62	---NA---
XLOC_071146	-3.54	—	—	nucl1_orysj ame: full=nucleolin 1 ame: full=protein nucleolin like 1
XLOC_039880	-5.92	—	—	predicted protein
XLOC_072929	-5.97	—	—	predicted protein
XLOC_071850	-6.07	—	-4.44	predicted protein
XLOC_090738	-6.14	—	—	predicted protein
XLOC_056048	-4.88	—	—	PREDICTED: uncharacterized protein LOC100830818
XLOC_002739	-4.30	—	—	protein
XLOC_094886	-4.63	—	—	protein
XLOC_049284	-5.39	—	—	protein
XLOC_041525	-5.65	—	—	protein
XLOC_045462	-5.92	—	—	protein
XLOC_040319	-6.52	—	—	protein
XLOC_024380	-5.16	—	—	Protein H2A.5

**Gene Id**	**Log$_2$(fold change）**			**Description**
	**XZ5**	**XZ54**	**Tadmor**	
XLOC_053272	-4.09	—	—	protein set

— represents no change. NA represents unknown.

Table S6 DEG up-regulated in both of XZ5 and XZ54

Gene Id	Log_2(fold change）			Description
	XZ5	XZ54	Tadmor	
XLOC_073621	3.94759	3.80	—	cortical cell-delineating protein

— represents no change.

Table S7 DEGs down-regulated in both of XZ5 and XZ54

Gene Id	Log_2(fold change）			Description
	XZ5	XZ54	Tadmor	
XLOC_089282	-2.73	-2.84	-4.62	caffeic acid o-methyltransferase
XLOC_036698	-5.16	-4.21	—	histone h2a
XLOC_092820	-4.58	-4.65	—	---NA---
XLOC_071561	-5.07	-6.02	-4.86	---NA---
XLOC_067168	-5.83	-4.31	-3.51	---NA---
XLOC_049544	-6.32	-5.01	—	---NA---
XLOC_090528	-5.85	-5.15	-4.77	predicted protein
XLOC_093109	-5.79	-6.07	-5.33	predicted protein

— represents no change. NA represents unknown.

Table S8 DEGs only up- or down-regulated in Tadmor

Gene Id	Log$_2$(fold change）			Description
	XZ5	XZ54	Tadmor	
**Metabolism**				
XLOC_083676	——	—	-2.17	s-adenosylmethionine decarboxylase
XLOC_095268	—	—	-2.47	stem 28 kda glycoprotein
XLOC_034864	—	—	-2.80	triacylglycerol lipase
XLOC_082307	—	—	-3.21	n- acetylneuraminyllactose-wheat germ agglutinin isolectin complexes
XLOC_054295	—	—	-3.40	gdsl esterase lipase at4g28780-like
XLOC_073045	—	—	-4.18	gdsl esterase lipase
XLOC_073015	—	—	-4.22	arginine decarboxylase
XLOC_074328	—	—	-4.32	rare lipoprotein a like double-psi beta-barrel containing expressed
XLOC_027571	—	—	-6.38	glycosyltransferase protein 2-like
XLOC_090724	—	—	-6.68	snare-interacting protein keule
XLOC_089757	—	—	-9.17	caffeic acid 3-o-methyltransferase
XLOC_037002	—	—	-5.07	low-molecular-weight cysteine-rich protein lcr69 precursor
**Stress defense**				
XLOC_084723	—	—	4.89	dhn4
XLOC_051828	—	—	2.74	dehydration responsive protein
XLOC_034696	—	—	-3.41	lipid-transfer protein dir1-like
XLOC_031430	—	—	-4.57	pathogenesis-related maize seed protein
XLOC_070385	—	—	-5.83	stress responsive protein
**Transcription**				
XLOC_022317	—	—	-3.60	nuclease s1
XLOC_041687	—	—	-4.82	transposon unclassified
XLOC_067227	—	—	-3.50	transposon protein precursor
XLOC_041304	—	—	3.03	is1 transposase partial
**Protein synthesis**				
XLOC_049399	—	—	2.14	23s ribosomal rna
**Cell growth**				
XLOC_063970	—	—	2.31	cell wall-associated hydrolase
XLOC_057886	—	—	3.56	cell wall-associated partial
XLOC_062551	—	—	-4.68	cortical cell-delineating protein
XLOC_059960	—	—	-6.00	cortical cell-delineating protein precursor
XLOC_083214	—	—	-5.34	keratin-associated protein 5-4-like
XLOC_091007	—	—	-4.63	root cap protein 1
XLOC_079375	—	—	-4.66	root cap protein 1
XLOC_018231	—	—	-4.11	root cap protein 1
XLOC_030829	—	—	-3.75	root cap protein 1
XLOC_001464	—	—	5.72	muscle actin
**Transporter**				
XLOC_034972	—	—	-5.50	mate efflux family protein
XLOC_030052	—	—	-5.06	organic cation transporter
**Unknown**				
XLOC_062789	—	—	8.72	predicted protein

Gene Id	Log$_2$(fold change)			Description
	XZ5	XZ54	Tadmor	
XLOC_057872	—	—	4.23	protein
XLOC_082234	—	—	3.06	hypothetical protein POPTR_1605s00200g
XLOC_026478	—	—	2.67	hypothetical protein
XLOC_023118	—	—	-4.37	hypothetical protein F775_03747
XLOC_092659	—	—	-4.10	---NA---
XLOC_031190	—	—	2.27	predicted protein

— represents no change. NA represents unknown.

Table S9 KEGG pathways of tolerance related root hair genes in response to drought stress

**Gene Id**	**Log_2 (fold change)**			**Pathway**
	**XZ5**	**XZ54**	**Tadmor**	
XLOC_013018	3.39	—	3.23	Arginine and proline metabolism
				Nitrogen metabolism
				Alanine, aspartate and glutamate metabolism
				Glyoxylate and dicarboxylate metabolism
				Glutathione metabolism
XLOC_013896	3.68	—	3.68	Selenocompound metabolism
				Sulfur metabolism
				Purine metabolism
XLOC_019100	4.85	—	-2.58	Phenylpropanoid biosynthesis
				Phenylalanine metabolism
XLOC_012656	2.5	—	2.72	Glyoxylate and dicarboxylate metabolism

— represents no change.

Text S1 Protein sequences used for alignment analysis in Figure 6.

>HvEXPB7 (NCBI accession number KR732966)

MAAAFSSYSVAAAFLCLLAANGCCGCPWFLPATFCPEPTPDPIPTPDPAPATPPPALATPSPGSGSGSA
NGSMGGWLDARATWYGAPDGAGPLDNGGACGFKNVNLPPFNAMTSCGNEPIFKDGKGCGSCYQ
IRCVGKVHPACSGDPETVIITDMNYYPVARYHFDLSGTAFGAMAKDGRNDELRHAGIIDMQFKRVP
CQYTGLSVTFHVEKGSNPNYLAILVEYGNGDGDVAQVDLMDGGEPTVSWRPMRESWGSIWRLDT
RRPLRGPFSLRVTNGSGRSLVADQVIPADWQPDTVYSSDVQFDE

>OsEXPB7 (UniProtKB accession number Q9LD07)

MAGRSRRRSFWSVGVAAALLCLLAAHGCSAKHHKPKPTPGGISGNASSSSSNSSTPSIPPPVAPTPT
APTPPIPSPGTGSSNGSSGGGGGGWLNARATWYGAPNGAGPDDNGGACGFKNVNLPPFSAMTSC
GNEPLFKDGKGCGSCYQIRCVGHPACSGLPETVIITDMNYYPVSLYHFDLSGTAFGAMAKDNRND
ELRHAGIIDIQFRRVPCQYPGLTVTFHVEQGSNPVYMAILVEYENGDGDVVQVDLMESRYSTGGVD
GTPTGVWTPMRESWGSIWRLDTNHPLQGPFSLRITNESGKTLIADQVIPADWQPNTVYSSIVQFD

>ZmEXPB7 (NCBI accession number AAK56130.1)

MATTLSSTVVVALGAPLFLLLVTCGSCARPVSFNASDLTADPGWDAARATWYGAPTGAGPDDDGG
ACGFKNVNLPPFSAMTSCGNEPLFKDGKGCGSCYQIRCQNHPACSGNPETVIITDMNYYPVAKYHF
DLSGTAFGAMAKPGRNDELRHAGIIDIQFKRVPCNYPGQKVTFHVEEGSNPVYFAVLVEFEDGDGD
AVQVDLMEANSASWTPMRESWGSIWRLDSGHRLTAPFSLRITNESGKTLVADHVIPANWVPNTYY
RSIVQY

>TaEXPB7 (NCBI accession number AAS48884.1)

MAGLLSVKAFALAAVLAAAYVSCAAAEQPGAADLDASAVSYSSAWLPARATWYGAPNGAGPDD
NGGACGFKHVNQYPFSSMTSCGNQPLFKDGKGCGSCNQIRCSGDKSCSGKIETVMITDMNYYPVA
QYHFDLSGTAFGALAKSGLNEKLRHSGIIDIQFRRVPCNFPGLKINFHVVDGSNAVYLAVLVEYED
MDGDVIQVDMKEANSGSWTPMRESWGSIWRMDSNHRLQGPFSMRITSDSGKKLVANNVIPANWR
PNTDYRSFVQFS

Text S2 Protein sequences used for phylogenetic tree analysis in Figure 7a.

>HvEXPB7 (NCBI accession number KR732966)
MAAAFSSYSVAAAFLCLLAANGCCGCPWFLPATFCPEPTPDPIPTPDPAPATPPPALATPSPGSGSGSA
NGSMGGWLDARATWYGAPDGAGPLDNGGACGFKNVNLPPFNAMTSCGNEPIFKDGKGCGSCYQ
IRCVGKVHPACSGDPETVIITDMNYYPVARYHFDLSGTAFGAMAKDGRNDELRHAGIIDMQFKRVP
CQYTGLSVTFHVEKGSNPNYLAILVEYGNGDGDVAQVDLMDGGEPTVSWRPMRESWGSIWRLDT
RRPLRGPFSLRVTNGSGRSLVADQVIPADWQPDTVYSSDVQFDE

>TaEXPA1 (NCBI accession number AAR27327.1)
MAAAGALFFLFSSFCLLARQAAAGGYGGWQSAHATFYDGGDASGTMGGACGYGNLYSTGYGTN
TAALSTALFNDGAACGSCYELRCDNAGSSCRPGSILVTATNFCPPNYGLPSDDGGWCNPPRPHFDM
AEPAFLHIAQHRAGIVPVPYRRVPCVKKGGIRFTINGHSYFNLVLVTNVAGAGDARSVSIKGTRTGW
QAMSRNWGMNWQSNTFLDGQCLSFRVTSSDGRTVTSNAAAPAGWHFGQTFEGAQF

>TaEXPA2 (NCBI accession number AAS48871.1)
METRRPAVSTVSVGAVVLILAWSPAAAVASGGWMDAHATFYGDETGAETMQGACGYGNLFQQGY
GLDTTALSVALFSDGWSCGGCYEIQCHGDPHCKPGGAQVTVTATNLCPANYSKPYENWCNPPLKH
FDLSKPMFLRLVTDFHVGIIPVQYRRVPCAKKGGIRIEMTGNQYWVGVLVFNVAGPGEVKVLAVK
GAKDGQWRNMKRNWGQIWDGHVQNLVGQGLSFRVVASDGRSVVLDGVVPASWTIGQSFEGKG
QF

>TaEXPA3 (NCBI accession number AAS48872.1)
MASANALLLLFSAAFCFLARRAAGGYGSWQSAHATFYGGGDASGTMGGACGYGNLYSSGYGTN
AAALSTALFNDGAACGSCYELKCQEVSSSCLPGSITITATNLCPPNYALPNDERGWCNPPRAHFDM
AEPAYLQIGIYRAGIVAVAYRRVPCVKKGGIRFTINGHSYFNLVLVTNVAGAGDVQSVAIKGYSTGW
QAMARIWGQNWQSNADLDGQSFSFRVTFSDGRTVTSNNAAPAGWSFGQTFEGAQF

>TaEXPA4 (NCBI accession number AAS48873.1)
METPSLLLFTTLLAFAFKASVAQWTPAFATFYGGSDASDTMGGACGYGNLYNAGYGVNSAALSTA
LFNNGASCGMCFTITCDASKTPSCKQGTSITITATNFCPPNYALASDNGGWCNPPRQHFDMSQPAW
ETIAVYQAGIVPVNYRRVPYQRSGGMRFTINGNDYFELVTVANVGGSGVVSQMWIKGFKTDWMV
MSRNWGASWQSNAYLNSQSISFRVQTDDGRVITADNVAPYNWWFGGTYTSWQQF

>TaEXPA5 (NCBI accession number AAS48874.1)
MAVIKMLLLPALFALLLRAASAAQWTPAHATFYGGGDASGTMGGACGYGNLYGAGYGTQTTALS
TALFNNGASCGACFTIACDTRKSRMCKPGTSITVTATNFCPPNYALAGDNGGWCNPPRQHFDMAQ
PAWETIAVYKAGIVPVNHRRVRCQRSGGIRFTINGHSYFELVLVTNVGGSGGVAQMWIKGSRTNW
MEMSRNWGANWQSNAKLDGQSLSFRVKSDDGRVVTANDVAPPGWWFGGTYTSGAQLY

>TaEXPA6 (NCBI accession number AAS48875.1)
MAAGMRFLQLFAAVLAFCFVQARSDYWHQAYATFYGGADGAETMGGACGYDNLYAAGYGLNN
AALSTVLFNNGLSCGQCYLITCDTSKSNMCKPGTSITVSATNLCPPNWALANDNGGWCNPPREHF
DMSQPAWENLAIYRAGIVPVLYQRVACQRQGGLRFTMSGFNYFELVLVTNIAMSGSIRSMSVKGTN
TAWITMSQNWGANWQCLAGLKGQALSFGITSSGGQYKVFQDVVPAWWLFGQTFSTWQQFDY

>TaEXPA7 (NCBI accession number AAS48876.1)
MAPPLILLVVFLLPALAAGHQHPSSYGSSALSEWRNAKSSYFAADPGDAIGGACGFGDLGKHGYG
MATVGLSTALFERGAACGGCYEVKCVEDLKYCLPGTSIVVTATNFCPPNYGFPADAGGVCNPPNH
HFLLPIQAFEKIALWKAGVMPIQYRRVKCLREGGVRFSVSGKSFFFTVLISNVGGAGDVRSVKIKGT
ESGWLDMGRNWGQIWHINLDLTGQPVSFELTSSDGTTMTNFNVVPKDWEFGKTYTGKQFLL

>TaEXPA8 (NCBI accession number AAS48877.1)
MEFLGLLALAIAAMVGVVAGDDSTWSNGRATFYGGNDASGTMGGACGYGNMFSAGYGTNTAAL
STALFNNGQSCGACFEIRCAGSGSCLPGSAVVTATNLCPANYALPNNEGGWCNPPQSHFDLAEPMF
TKIAQARAGVVPVQYRRVVCVKTSGIRFTITGHSYFNLVLITNVAGAGDLTAVYVKSPSTGWLTMS
HNWGANWQNGAMLNGQPLSFRVTTSDGRTTTSNNVAPSGWSFGQTYAGSQF
>TaEXPA9 (NCBI accession number AAS48878.1)
MAAAMPPSLPALLAALLLLVTLSPAAARVPGVYTGGDWQSAHATFYGGTDASGTMGGACGYGNL
YSQGYGVNNAALSTALFNEGQRCGACFEIRCVNQPGWAWCLPGRPSIVVTATNFCPPNYALPSDDG
GWCKPPRPHFDLAMPMFLHMAQYRAGIVPVSYRRVACRKSGGVRFTINGFRYFNLVLITNVAGAG
DLVRASVKGSSTGWMPMSRNWGQNWQSNAILVGQALSFRVTASDRRTSTSWNAAPQNWRFGRT
FEGKNLRV
>TaEXPB1 (NCBI accession number AAS48879.1)
MAPLSSKAVALVALFSLLVTYAAGAGNFNDSAFTADPNWEDARATWYGAPTGAGPDDDGGACGF
KNTNQYPFSSMTSCGNEPIFKDGKGCGSCYQMRCTNDQSCSGNPETVVITDMNYYPVAKYHFDLS
STAFGAMAKPGLSEKLRHSGIIDIQFKRVPCEFPGLKVTFHVEQGSNPVYFAVLVEYEDGDGDVVQ
VDLMEANSGTWTPMRESWGSIWRLDSGHRLQAPFSMRITNESGKTLVADKVIPANWAPSTFYRSIV
QYS
>TaEXPB2 (NCBI accession number AAS48880.1)
MGSLPLAVLAALLCCFLAVGGGAVELNTTDPSPFDVDLNATDATKYWGPWTPARATWYGQPNGA
GPDDNGGACGFKHTNQYPFASMTSCGNQPLFKDGKGCGSCYKIRCRKDMSCSGRTETVIITDMNY
YPVAPFHFDLSGTAFGRLAKPGLNDRLRHSGIIDIEFTRVPCEFPGLKIGFHVEEYSNPVYFAVLVEY
EDGDGDVVQVDLMESRGPGGGKWTRMRESWGSVWRLDSNHRLQAPFSIRIRNESGKTLVANKVI
PANWRPNTFYRSFVQYS
>TaEXPB3 (NCBI accession number AAS48881.1)
MAPLSSKAVALVALFSLLVTYAAGAGNFNDSAFTADPNWEDARATWYGAPTGAGPDDDGGACGF
KNTNQYPFSSMTSCGNEPIFKDGKGCGSCYQIRCTNDQSCSGNPETVVITDMNYYPVAKYHFDLSG
TAFGAMAKPGLSEKLRHSGIIDIQFKRVPCEFPGLKVTFHVEQGSNPVYFAVLVEYEDGDGDVVQV
DLMEANSGTWTPMRESWGSIWRLDSGHRLQAPFSMRITNESGKSLVADKVIPANWAPSTFYRSIVQ
YS
>TaEXPB4 (NCBI accession number AAS48882.1)
MASSSSSVLLVAAVLAAVVCGAHGIAKVPPGPNITASPTSYGNKWLDAKTTWYGKPTGAGPKDNG
GACGYKEVDKAPFHGMTSCGNIPIFKDGRGCGSCFELKCTKPEACSGEPTMVTITDKNEEPIAPYH
FDLSGHAFGSMAKKGEEQKLRDAGEVEIKFRRVKCKYPAGTKVNFHVEKSSNENYLALVIKFLQG
DGDVVGVDIKQKGEDKWTELNESWGAVWRIDTPHKLIGPFSVRYTTEGGTKTVVDDVIPKGWKP
DTSYEAKGGY
>TaEXPB5 (NCBI accession number AAS48883.1)
MGSVSYVPAAAVLAALVSGGACIPSVPPGPNITTNYNNQWLPAKATWYGKPTGSGPKDNGGACGI
KDVNLAPYNGMIACGNVPIFKDGKGCGSCHEIKCQKPSPCSDKPVTIFITDKNYEPIAPYHIGLSGT
AFGAMATPGKEQTLRSFGELELQFRRVRCKYAPGTKITFHVEKGSNPNYLAVLVKFVSDDGDVVQ
MDIQESKSPAWIPLTLSWGAIWRWDGAKPLKGPFSIRVTSEPGKKLIAKDVIPANWKADTVYTSNV
QF
>TaEXPB7 (NCBI accession number AAS48884.1)
MAGLLSVKAFALAAVLAAAYVSCAAAEQPGAADLDASAVSYSSAWLPARATWYGAPNGAGPDD
NGGACGFKHVNQYPFSSMTSCGNQPLFKDGKGCGSCNQIRCSGDKSCSGKIETVMITDMNYYPVA
QYHFDLSGTAFGALAKSGLNEKLRHSGIIDIQFRRVPCNFPGLKINFHVVDGSNAVYLAVLVEYED
MDGDVIQVDMKEANSGSWTPMRESWGSIWRMDSNHRLQGPFSMRITSDSGKKLVANNVIPANWR

PNTDYRSFVQFS

>TaEXPB8 (NCBI accession number AAS48885.1)

MAGVSTNAIALVLVTLLSVLVTSVRSAANYDTAAARSYNSGWLPAKATWYGAPTGAGPMDNGGA
CGFKNVNKYPFSSMTSCGNEPLFDGGAGCGSCYEIRCIAANNPSCSGQPRTVVITDMNYYPVARYH
FELSGTAFGAMAKYGLNDKLRHAGIIDMQFRRVRCNFPGMKVTFHVQRGSNPNYLAVLVEYANID
GTVVRMELMQTRNGRPTGSWEPMRRSWGSIWRMDTSRPLQGPFSMRITSDSGKTLVANNVIPAY
WRPDNAYWSNVQFY

>TaEXPB9 (NCBI accession number AAS48886.1)

MAKTCTLALLGALLVLSLLVSPIACSRKLAKVGGHDKSAPVKGHKNQTTTNPSSSAAYGGGWLPA
GATYYGNPNGDGSDGGACGYQTAVGHRPFSSMIAAGSSPLFMAGKGCGACYDVKCTSNSACSGK
PVNIVITDLSPGNLYPGEPCHFDMSGTALGAMAKPGMADKLRAGGVIRMQYKRVPCKYPGVNIAF
RVDQGSNPFYFKTLIEFEDDDGDLKAVALKEAGSGAWTPMAQDWGALWRLNEGRQLRAPFSLRL
TSDSGRKLVVSNVIPANWKAGATYRSLVNYP

>TaEXPB10 (NCBI accession number AAS48887.1)

MAGVSSNAIAFVALLSVLFTGVRSAVNYDTAVARSYNSGWLPAKATWYGAPNGAGPDDNGGACG
FKNVNQYPISSMGACGNEPIFAGGEGCGMCYEIKCDYSNNPSCSGQPRRIVITDMNYYPVARYHLD
LSGTAFGSMARYGLNDRLRHAGIIDMQFRRVPCNFPGMKVTFHVQRGSNPNYLAVLVEYANVDGT
VVRMELMQTINGRPTGYYQDMRRSWGSIWRMDTNRPLQGPFAIRITSDTGKTLVANNAIPAYWQP
DHAYWSNLQFY

>OsEXPA1 (UniProtKB accession number Q7XWU8)

MGGACGYGDLYSTGYGTNTAALSTVLFNDGASCGQCYRIMCDYQADRRFCISGTSVTITATNLCPP
NYALPNDAGGWCNPPRQHFDMAEPAWLKIGVYVGGIVPVMYQRVPCAKQGGVRFTINGRDYFEL
VLVSNVGGVGSIQSVSIKGSRTGWMAMSRNWGVNWQSNAYLDGQSLSFKVTSSDGQTLTFLDVAP
AGWTFGQTFSTSQQFS

>OsEXPA2 (UniProtKB accession number Q40636)

MASRSSALLLLFSAFCFLARRAAADYGSWQSAHATFYGGGDASGTMGGACGYGNLYSTGYGTNT
AALSTVLFNDGAACGSCYELRCDNDGQWCLPGSVTVTATNLCPPNYALPNDDGGWCNPPRPHFD
MAEPAFLQIGVYRAGIVPVSYRRVPCVKKGGIRFTINGHSYFNLVLVTNVAGPGDVQSVSIKGSSTG
WQPMSRNWGQNWQSNSYLDGQSLSFQVAVSDGRTVTSNNVVPAGWQFGQTFEGGQF

>OsEXPA3 (UniProtKB accession number Q40637)

MLSGMEKQPAMLLVLVTLCAFACKXSVAQSAFATFYGGKDGSCTMGGACGYGNLYNAGYGLYN
AALSSALFNDGAMCGACYTITCDTSQTKWCKPGGNSITITATNLCPPNWALPSNSGGWCNPPLQHF
DMSQPAWENIAVYQAGIVPVNYKRVPCQRSGGIRFAISGHDYFELVTVTNVGGSGVVAQMSIKGSN
TGWMAMSRNWGANWQSNAYLAGQSLSFIVQLDDGRKVTAWNVAPSNWFFGATYSTSWVQF

>OsEXPA4 (UniProtKB accession number Q0DHB7)

MAIAGVLFLLFLARQASAAGYGGWQSAHATFYGGGDASGTMGGACGYGNLYSQGYGTNTAALS
TALFNDGAACGSCYELRCDNAGSSCLPGSITVTATNFCPPNYGLPSDDGGWCNPPRPHFDMAEPAF
LHIAQYRAGIVPVSFRRVPCVKKGGVRFTVNGHSYFNLVLVTNVAGAGDVRSVSIKGSRTGWQPM
SRNWGQNWQSNAFLDGQSLSFQVTASDGRTVTSNNVAHPGWQFGQTFEGGQF

>OsEXPA5 (UniProtKB accession number Q6ZGU9)

MSSRRDVLAVVLVAALLPPALSRGLWLGHHGLGHGHGRWRAPHVGGHGQGQGPQQHAPLGGGG
WSSAHATFYGGGDASGTMGGACGYGNLYSQGYGTNTAALSTALFNNGLSCGACFEVRCDAGGG
GSHSCLPGSVVVTATNFCPPNNALPSDDGGWCNPPRAHFDMSQPVFQRIALFKAGIVPVSYRRVAC
QKKGGIRFTINGHSYFNLVLVTNVGGAGDVHAVAVKSERSAAWQALSRNWGQNWQSAALLDGQ
ALSFRVTTGDGRSVVSNNAVPRGWSFGQTFSGAQFN

>OsEXPA6 (UniProtKB accession number Q9M4X7)

MAPPLLLLLASLLLVAARRALGLGLGQWQPGHATFYGGGDASGTMGGACGYGNLYSQGYGTSTA
ALSTALFNRGLSCGSCYELRCAGDHRRSCLPGGATVTVTATNFCPPNYALPSDGGGWCNPPRRHFD
LAEPAFLRIARHAAGIVPVSFRRVACARKGGVRFTVNGHAYFNLVLVTNVGGAGDVRSLAVXGSGS
GSRVGGRWQPMSRNWGQNWQSNAYLDGKALSFRVTAGDGRSLTCADVAPAGWQFGQTFEGRQF

>OsEXPA7 (UniProtKB accession number Q852A1)

MSPAPRVLVLVVATVVALQVSPAAGRIPGAYGGGEWQSAHATFYGGSDASGTMGGACGYGNLYSQ
GYGVNNAALSTALFNSGQSCGACFEIKCVNQPGWEWCHPGSPSILITATNFCPPNYALPSDNGGWC
NPPRPHFDLAMPMFLHIAEYRAGIVPVSYRRVPCRKKGGVRFTINGFRYFNLVLITNVAGAGDIVRA
SVKGTSTGWMPMSRNWGQNWQSNSVLVGQALSFRVTGSDRRTSTSWNAAPAGWHFGQTFEGKN
FRV

>OsEXPA8 (UniProtKB accession number Q9XHX0)

MAAARMLVLLASLCALLLTASAAKWTPAFATFYGGSDASGTMGGACGYGDLYGAGYGTRTAALS
TALFNGGASCGACFTIACDTRKTQWCKPGTSITVTATNFCPPNYALSGDAGGWCNPPRRHFDMSQP
AWETIAVYRAGIVPVNYRRVPCQRSGGIRFAVNGHSYFELVLVTNVGGSGAVAQMWIKGSGTGWM
AMSRNWGANWQSNARLDGQALSFRVQADDGRVVTAADVAPAGWSFGATYTSSAQFY

>OsEXPA9 (UniProtKB accession number Q4PR53)

MEKKLLVVLFLSLCCASRLRGEAAQQWTSATATFYGGSDASGTMGGSCGYGNMYSAGYGTNTTA
LSSALYGDGASCGACYLVTCDASATRWCKNGTSVTVTATNYCPPNYSESGDAGGWCNPPRRHFD
MSQPAWEAIAVYSSGIVPVRYARTPCRRVGGIRFGIAGHDYYELVLVTNVAGSGAVAAAWVKGSGT
EWLSMSRNWGENWQSNAYLTGQALSFRVQADDGGVVTAYDVAPANWQFGSTYQSDVNFSY

>OsEXPA10 (UniProtKB accession number Q7XUD0)

MAPCLLLVLFLLPALATGHQHPSTLGSSALSEWRSAKASYYAADPEDAIGGACGFGDLGKHGYGM
ATVGLSTALFERGAACGGCYEVKCVDDLKYCLPGTSIVVTATNFCAPNFGLPADAGGVCNPPNHH
FLLPIQSFEKIALWKAGVMPIQYRRVNCLRDGGVRFAVAGRSFFLTVLISNVGGAGDVRSVKIKGTE
SGWLSMGRNWGQIWHINSDFRGQPLSFELTSSDGKTLTNYNVVPKEWDFGKTYTGKQFLL

>OsEXPA11 (UniProtKB accession number Q4PNY1)

MELLRLLAVAAVAAMAAEVAAGGDSGWSSGSATFYGGSDASGTMGGACGYGNLYSAGYGTSTAA
LSTALFNNGQSCGACFEVRCGGGGSCLAGTVAVTATNLCPPNYALAGDAGGWCNPPRPHFDMAEP
AFTRIAQARAGVVPVQYRRVACAKQGGIRFTITGHSYFNLVLVTNVGGAGDVTAVSVKGSRSGWQ
AMSHNWGANWQNGANLDGQPLSFRVTASDGRTVTSDNVAPSGWSFGQTFSGGQF

>OsEXPA12 (UniProtKB accession number Q7G6Z2)

MARSAFFFHCVAAVAACIAATAAALSGTATFYGGSDASGTMGGACGYGNLYSTGYGTNTAALSSA
LFNDGAACGECYQITCDQSNSKWCKAGTSVTITATNLCPPDYSKPSNDGGWCNPPRQHFDMAQPA
WEQIGVYRGGIVPVNFQRVSCTRKGGVRFTINGNSYFELVLITNVGGPGSIKSVQIKGTKTGWVTM
SRNWGANWQANNYLNNQAISFSVTSTAGKTLVFEDVAPSNWQFGQTFTSGVQFY

>OsEXPA13 (UniProtKB accession number Q4PR52)

MAGVARMLAAVVCAIMPAAAMAAGGVGALEPSGWVRAHATFYGGADASGTMGGACGYGNLYA
QGYGTRTAALSTALFNDGLACGQCYKLVCDRKTDRTWCKPGVSVTITATNFCPPNWDLPSDSGGW
CNPPRPHFDMAQPAWEKIGIYRGGIIPVIYQRVPCMKKGGVRFTINGHDYFQLVLLTNVGAAGSIKA
MDVKGSKSPDWMAMAHNWGAQWHSLAYLTGQGLSFRVTITDGQTLVFPNVVRPGWRFGQTFAS
NIQFK

>OsEXPA14 (UniProtKB accession number Q4PR51)

MASSPRAFALVFFAIAAVGCTQLTTADDAAPPVWQKAHATFYGGADASGTMGGGCGYGDLYSQG
YGTRNAALSTALFNDGASCGQCYKIACDRKRAPQWCKPGVTVTITATNFCPPNWDLPSDNGGWC
NPPRPHFDMAQPAWEKIGIYSAGIIPVIYQRVPCIKKGGVRFTINGHDYFNLVLVTNVATTGSIKSMD
IMGSNSTDWMPMVRNWGANWHSLSYLTGQTLSFRVTNMDGQTLVFKNIVPSGWKFGQTFTSKLQ

FK
>OsEXPA15 (UniProtKB accession number Q4PR50)
MAMWKKKKTPSILPLVVVIAAASLIAPTTAGWSSGTATFYGGSDASGTMGGACGYGNLYWSGYG
TNTAALSSALFNDGASCGQCYQIACDHQAEPRWCLQGRTVTITGTNLCPPNYALSSNDGGWCNPP
RTHFDMAEPAWLQIGIYKAGIVPVLYQRVPCVKQGGVRFTMGGFNYFELVLISNVAGSGSIQSVWV
KGPNTDRMPLSRNWGANWQSHAGLVGQTLTFGVTSTGGQTLVFQNIVPAWWKFGQSFSSNLQFS
Y
>OsEXPA16 (UniProtKB accession number Q69XV9)
MSSVLLFLLLLLLSGVSLSGCIRLGSGGYEEWRMGSATYIKESLGHPLNDGGGACGYGDLDIFRYG
RYTAGVSGALFGRGSACGGCYEVRCVNHVLWCLRGSPTVVVTATDFCAPNLGLSDDYGGWCNFP
KEHFEMSEAAFLRVAKAKADIVPVQFRRVSCDRAGGMRFTITGGASFLQVLITNVAADGEVAAVK
VKGSRTGWIPMGRNWGQNWQCDADLRGQPLSFEVTGGRGRTVVAYSVAPPDWMFAQTFEGKQF
VE
>OsEXPA17 (UniProtKB accession number Q4PR49)
MASSWNNPAIFLAAALAVATAAQVVTAGFTTDLYWQQQPAPGAVTPYKTSDWHDGSATFYGDPSG
MGDDFGGACGYVSNDIVSLYSTKTAALSTPLFADGNGCGQCYELRCVKSPWCNPGSPSVVITGTN
LCPPNWYLPNDDGGWCNPPRHHFDMAPPSFLKLAQRVAGIVPVQYRRVPCQRTGGVRFCLQGNH
YWLLLYVMNVGGAGDVSSLSVKTSGGGGAWIQAAHNWGITYQVFAALDNSDGLTVKLTTYSTPQ
QTIIVSDAISPWWITGLCYQGSNNFY
>OsEXPA18 (UniProtKB accession number Q4PR48)
MGNIVLQLLAILALCIAPARSGWLQGTATFYGGADGSGTMGGACGYGNLYDQGYGINNAALSTPL
FNNGASCGQCYLIICNYDKAPSGCRMGTAITVTGTNFCPPNYDLPYGGWCNTTRPHFDMSQPAWE
NIGIYSAGIVPILYQQVKCWRSGGVRFTITGLNYFELVLVTNMAGSGSIASMSVKGSSTGWIQMSRN
WGANWQCLAGLAGQALSFTVTSTGGQTIVFDSVVPAGWSFGQTFSTYQQFDY
>OsEXPA19 (UniProtKB accession number Q7G6Z5)
MGNIFLQLLAVVALCIAPARSDWLPGTATFYGGADGSGTMGGACGYGNLYDQGYGINNAALSTPL
FNDGASCGQCYLIICDYSKAPDWCKLGKAITVTGTNYCPPNYDLPYGGWCNATRPHFDMSQPAW
ENIGIYNAGIIPILYQQVKCWRYGGVRFTINGFNYFELVLVTNMAGSGSIASMSVKGSCTGWIQMTR
NWGANWQCLAGLAGQALSFNVTSTGGQTIVFDDAVPAGWSFGQTFSTYHQFDY
>OsEXPA20 (UniProtKB accession number Q10RK1)
MGNILLQLLAVVALCIAPARSDWLPGTATFYGGADGSGTMGGACGYGNLYDQRYGINNAALSTPL
FNDGASCGQCYLIICDYGKAPDWCKLGKAITVTGTNYGGWCNATRPYFDMSQPAWENIGIYSAGI
VPILYQQVKCWRYGGVRFIINGFNYFELVLVTNMPGSGSIVSMSVKGSCTGWIQMTRNWGANWQ
CLAGLAGQALSFNVTSTGGQTIVFDDAVPAGWSFGQTFSTYHQFDY
>OsEXPA21 (UniProtKB accession number Q10KN4)
MAPPSLPILLVLLSLSSSLSSSSAAAAGRWTDAHATFYGGADASGTMGGACGYGNTYGQGYGTDT
AALSAVMFGDGLSCGACFELRCGGGGGGDRRGCLPPAAGKSIVVTATDLCPANHALPGDRGGWC
NPPLHHFDLSQPAFLRIARFQSGIVPVSYRRVACRRKGGMRFTINGHSYFNLVLVSNVGGAGDVHA
VAVKAGGGRKARWQAMARNWGQNWQSGALLDGQALSFTVTTGDRRSVVSYNVAPAGWAFGQT
FTGRQFT
>OsEXPA22 (UniProtKB accession number Q4PR44)
MAPARPFALLFLAVTVGFVLLTAADDSANATATTTTAMAPSSSTDDAAPPVWLKAHATFYGGADA
SGTMGGACGYGDLYSQGYGTRNAALSTALFNDGASCGQCYKIACDRKRAPQWCRPGVTVTITAT
NFCPPNWDLPSDNGGWCNPPRPHFDMAQPAWEKIGIYRAGIIPVIYQRVPCVKKGGVRFTINGHDY
FNLVLVTNVATTGLIKSMDVMGSNSTDWLPMVRNWGANWHSLSYLTGQMLSFRVTNMDGQTLV
FRNIVPSGWKFGQTFASKLQFK

>OsEXPA23 (UniProtKB accession number Q4PR43)
MAPARAFVLVLLAVASASTAAANTATTTPTNPVAAPTQWQKAHATFYGGADASGTMGGACGYGN
LYSQGYGTRNAALSTALFNDGASCGQCYKIACDRKRAPQWCKPGVTVTITATNFCPPNWNLPSDN
GGWCNPPRPHFDMAQPAWEKIGVYSAGIIPVIYQRVPCVKKGGLRFTINGHDYFQLVLVTNVAAAG
SIKSMEVMGSNTADWMPMARNWGAQWHSLAYLTGQGLSFRVTNTDDQTLVFTNVVPPGWKFGQ
TFASKLQFK
>OsEXPA24 (UniProtKB accession number Q4PR42)
MADMAPARALALVLLAVAVGSALMAAAQDAPSPPTPMAPSPSTDETPPVWLKAHATFYGGADAS
GTMGGACGYVDLYSQGYGTRNAALSTALFNDGASCGQCYKIACDRKRAPQWCKPGVTVTVTAT
NFCPPNWNLPSDNGGWCNPPRPHFDMAQPAWEKIGIYRAGIIPVMYQRVPCVKKGGVRFTINGHD
YFNLVLVTNVATTGSIKSMDIMGSNSTDWMPMVRNWGANWHSLSYLTGQMLSFRVTNMDGQTL
VFRNIVPSGWKFGQTFASKLQFK
>OsEXPA25 (UniProtKB accession number Q4PR41)
MEYAILFATSLVITVLAASGFAPAHGWNKGTATFYGGADASGTMGGACGYGNLYTAGYGTNTAAL
SSVLFNDGWSCGQCYLIMCDAAATPQWCRAGAAVTITATNLCPPNWALPSNSGGWCNPPRPHFDM
AEPAWLQIGIYKAGIIPVLYQQVKCWRQGGIRFTMGGFNFFELVLVSNVAGSGSVRSVSVKGGSTG
WITLNRNWGANWQCNSGLVGQALSFAVTSTGGQTLYIYNVVPSWWSFGMTFTSNQQFSY
>OsEXPA26 (UniProtKB accession number Q2QP13)
MAPLPLLTTTSLLLFFFLASSFAADVVVAGGGGGGGGYDGGGDGEGGGGGDGEGGGGGGGAKM
PHVNHGRYKCGPWVDGHATFYGGRDASGTTEGGACGYKDADGYGAMTAAVSPALFDNGAGCG
ACYELKGDSGKTVVVTATNQAPPPVNGMKGEHFDLTMPAFLSIAEEKLGVVPVSYRKVACVRQGG
IKYTITGNPSYNMVMVKNVGGAGDVVKLTVKGTKRVKWTPLQRSWGQLWKTEANLTGESLTFRV
MTGDHRKATSWRVAPRDWTYDNTYQAKKNF
>OsEXPA27 (UniProtKB accession number Q7XE35)
MGAMAENLLVLCTILAARMALAAADDWIPATATFYGGNDGSGTMGGACGYGNLYDQGYGLENA
ALSTALFNDGAACGQCYLIVCDTDKAGRWCKPRGAVTVTATNLCPPNWALPSDGGGWCNPPRRH
FDMSQPAWERIGVYRAGIVPVLYRRVRCWRRGGVRFTVGGFDHFELVLVANVAGSGSVAAVSVRG
AGTGWLQMSRNWGANWQSLAGLAGQPLSFGVTTTGGQYILFQDVAPAGWKFGQTFSTSKQFDY
>OsEXPA28 (UniProtKB accession number Q4PR40)
MVIRFFAVLAAALCITSASAAAAGGWVSGTATFYGGKDASGTMGGACGYGNLYTQGYGVYNAAL
STALFNGGASCGQCYLIMCDASKTPEWCKAGTAVTITATNLCPPNWALANDDGGWCNPPRPHFDM
SQPAWETIGIYRAGIVPVLYQQVKCWRQGGVRFTVSGFNYFELVLITNVAGSGSVQAMSVKGSKTG
WIPLARNWGANWQCNSALVGQALSFRVTSTGGQTLQINSVVPEWWEFGTTFTSNQQFDY
>OsEXPA29 (UniProtKB accession number Q4PR39)
MARRGHVFAVVAFVSYALLAAASTTVEAFAASGWSKGTATFYGGSDASGTMGGACGYGNLYTQG
YGTRTAALSTALFDDGASCGQCYALTCDARADPRWCRAGASVTVTATNFCPPNYALPSDDGGWCN
PPRPHFDMAQPAWERIGVYRGGIVPVAFRRVPCRRRGGVRFTVAGRDYFELVLVTNVAAAGSVRS
MEVRGSRRGAGWMAMSRNWGANWQSLAYLDGQGLSFRVTATDGQTIVFAGVVPPSWRFGQTFA
STQQFM
>OsEXPA30 (UniProtKB accession number Q8W2X8)
MAAASSTTATTAILAAVIISLAGAATTVDAKFRAMQWTPAHATFYGDETASETMGGACGYGNLYAS
GYGTDTAALSTTLFKDGYGCGTCYQMRCVGTASCYRGSPAITVTATNLCPPNWAEDPDRGGGGW
CNPPRAHFDLSKPAFMRMADWRAGIVPVMYRRVPCARAGGLRFALQGNPYWLLAYVMNVAGAG
DVGDMWVKAGGGGGWVRMSHNWGASYQAFAQLGGQALSFKVTSYTTGQTILAAGVTPASWCF
GLTYQARVNFS
>OsEXPA31 (UniProtKB accession number Q75I75)

MAMSSRLALCLAVVAACAAGGAVADWSPATATFYGGSDGSGTMGGACGYGNLYDQGYGVDNAA
LSQALFNDGASCGQCYLIVCDTSRAPQWCKAGTAVTVTATNLCPPNWALPSDGGGWCNPPRPHFD
MSQPAWEQIGVYQAGIVPVLYQRVRCWRQGGVRFTVAGLNYFELVLITNVAGSGSVASAWIKGTN
TGWIQMSRNWGANWQSLAGLAGQALSFAVTTTGGQYLQFQDVAPAWWQFGQTFSTYQQFDY

>OsEXPA32 (UniProtKB accession number Q6YYW5)

MWCTWALGRVVLAVVFLVALAAGDAAPPKVHRNHGKFTAGPWKQAHATFYGGRDGSGTLDGAC
GYKDTSKEGYGVQTVAVSTPLFGAGAGCGACYEVKCVDSPDGCKVGAAPLVVTATNLCPPNPGQS
NDNGGWCNPPREHFDLSMPAFLQIAQEKAGIVPISYRRVPCVKVGGIRYTITGNPYFNLVMVSNVG
GAGDVAGLSVKGNKRVKWTPLKRNWGQEWQTSEVLTGESLTFRVMTGDHRKATSWHVLPPDWQ
FGVTYQATKNFN

>OsEXPA33 (UniProtKB accession number POC1Y4)

MAMPVVQVLLLCALAYQAVDAQWTPATATFYGGSDGAGTMGGACGYGNLYNAGYGLNNAALSS
ALFNDGAMCGACYTIACDTSQSTWCKPGTSITITATNLCPPNYAKKSDAGGWCNPPRKHFDMSQP
AWTSIAIYQAGIVPVNFKRVPCQKSXGIRFTISGRDYFELVTVFNVGGSGVVAQVSIKGSKTDWMA
MSRNWGQNWQSNAYLNTQSLSFKVKLDDAREVTVWNIAPSNWNFGTTYTSNINF

>OsEXPB1 (UniProtKB accession number Q40638)

MASSSLLLACVVVAAMVSAVSCGPPKVPPGPNITTSYGDKWLEAKATWYGAPKGAGPKDNGGAC
GYKDVDKAPFLGMNSCGNDPIFKDGKGCGSCFEIKCSKPEACSDKPALIHVTDMNDEPIAAYHFDL
SGLAFGAMAKDGKDEELRKAGIIDTQFRRVKCKYPADTKITFHIEKASNPNYLALLVKYVAGDGD
VVEVEIKEKGSEEWKALKESWGAIWRIDTPKPLKGPFSVRVTTEGGEKIIAEDAIPDGWKADSVYK
SNVQAK

>OsEXPB2 (UniProtKB accession number O24230)

MAGASAKVVAMLLSVLATYGFAAGVVYTNDWLPAKATWYGQPNGAGPDDNGGACGFKNTNQY
PFMSMTSCGNEPLFQDGKGCGACYQIRCTNNPSCSGQPRTVIITDMNYYPVARYHFDLSGTAFGAM
ARPGLNDQLRHAGIIDIQFRRVPCYHRGLYVNFHVEAGSNPVYLAVLVEFANKDGTVVQLDVMES
LPSGKPTRVWTPMRRSWGSIWRLDANHRLQGPFSLRMVSESGQTVIAHQVIPANWRANTNYGSK
VQFR

>OsEXPB3 (UniProtKB accession number Q336T5)

MAFSISKKAAVAALFSFLVVTCVAGARPGNFSASDFTADPNWEVARATWYGAPTGAGPDDDGGAC
GFKNTNQYPFSSMTSCGNEPIFKDGKGCGSCYQIRCVNHPACSGNPETVIITDMNYYPVSKYHFDL
SGTAFGAMAKPGQNDQLRHAGIIDIQFKRVPCNFPGLKVTFHVEEGSNPVYFAVLVEYEDGDGDV
VQVDLMEANSQSWTPMRESWGSIWRLDSNHRLTAPFSLRITNESGKQLVASQVIPANWAPMAVYR
SFVQYSS

>OsEXPB4 (UniProtKB accession number Q94LR4)

MGSLSSLAAAAVFLSLLAVGHCAAADFNATDADADFAGNGWDFNSSDAAVYWGPWTKARATWY
GQPNGAGPDDNGGACGFKHTNQYPFMSMTSCGNQPLFKDGKGCGSCYKIRCTKDQSCSGRSETV
IITDMNYYPVAPFHFDLSGTAFGRLAKPGLNDKLRHSGIIDIEFTRVPCEFPGLKIGFHVEEYSNPVYF
AVLVEYEDGDGDVVQVDLMESKTAHGPPTGKWTPMRESWGSIWRLDTNHRLQAPFSIRIRNESGK
TLVANNVIPANWRPNTFYRSFVQYS

>OsEXPB5 (UniProtKB accession number Q7XT39)

MVSRGTFVFAVLVALPILSLPVSGYEQNYTAGRRSTMSLGRGYGWSSGGATWYGGPQGDGSEGGA
CGYQSAVGQRPFSSMIAAGGPSLFKNGKGCGSCYQIKCTGNRACSGRPVTVVITDSCPGGVCLNEA
AHFDMSGTAFGAMANRGMGDRLRSAGVLKIQYKRVPCRFAMNVAFKVDAGSNPYYLAILVQYAN
GDGDLAAVHIMKARGGGGWKAMQQSWGATWRLNSNTGKPLSPPFSIRLTSGSGKVLVANNVIPSG
WQAGLTYRSTVNYAA

>OsEXPB6 (UniProtKB accession number Q7XCA7)

MAARMGSKVAAILAILSVLVVHGSCKGHPVNYNVSDASAYGSGWLPARATWYGAPTGAGPDDN
GGACGFKNVNQYPFSSMTSCGNEPIFKDGKGCGSCYQIRCNKDPSCSGNIETVIITDMNYYPVARY
HFDLSGTAFGAMAKPGLNDKLRHSGIIDIQFRRVPCNYPGLKINFHVEEGSNPVYFAVLVEYEDLDG
DVVQVDLMESKSAYGGATGVWTPMRESWGSIWRLDSNHRLQAPFSLRIRSDSGKTLVANNVIPAN
WSPNSNYRSIVQFS

>OsEXPB7 (UniProtKB accession number Q9LD07)

MAGRSRRRSFWSVGVAAALLCLLAAHGCSAKHHKPKPTPGGISGNASSSSSNSSTPSIPPPVAPTPT
APTPPIPSPGTGSSNGSSGGGGGGWLNARATWYGAPNGAGPDDNGGACGFKNVNLPPFSAMTSC
GNEPLFKDGKGCGSCYQIRCVGHPACSGLPETVIITDMNYYPVSLYHFDLSGTAFGAMAKDNRND
ELRHAGIIDIQFRRVPCQYPGLTVTFHVEQGSNPVYMAILVEYENGDGDVVQVDLMESRYSTGGVD
GTPTGVWTPMRESWGSIWRLDTNHPLQGPFSLRITNESGKTLIADQVIPADWQPNTVYSSIVQFD

>OsEXPB8 (UniProtKB accession number Q10T32)

MVSGDVGVVVYYLLLVLVVVQGCKGSSAVQGEGRWYNESEAIGGAAAWGNAKATWYGQPNGA
GAADNGGACGFKKVNQYPFMGMTSCGNQPLYKGGKGCGSCYRVRCNRNPACSGNAQTVAITDM
NYFPLSQYHFDLSGIAFGRLAKPGRADDLRRAGIIDVQFARVPCEFPGLKVGFHVEEGSSPVYLAVL
VEYENGDGDVAQVDLKEAGAGGGRWTPMRESWGSVWRLDSNHRLRAPFSIRIRSDSGKTLVAPD
VIPLNWTPNTFYRSFVQYSS

>OsEXPB9 (UniProtKB accession number Q7XCG7)

MGSLTTNIVLAVAVVAALVGGGSCGPPKVPPGPNITTNYNAPWLPARATWYGQPYGSGSTDNGGA
CGIKNVNLPPYNGMISCGNVPIFKDGRGCGSCYEVKCEQPAACSKQPVTVFITDMNYEPISAYHFD
FSGKAFGAMACPGKETELRKAGIIDMQFRRVRCKYPGGQKVTFHVEKGSNPNYLAVLVKFVADDG
DVIQMDLQEAGLPAWRPMKLSWGAIWRMDTATPLKAPFSIRVTTESGKSLIAKDVIPVNWMPDAI
YVSNVQFY

>OsEXPB10 (UniProtKB accession number Q8H7T4)

MASSCLLLACVVAAAMVSAVSCGPPKVPPGPNITAAYGKQWLEARGTWYGKPKGAGPDDNGGA
CGYKDIDKAPFLGMNSCGNDPIFKDGKGCGSCFEVKCSKPEACSDKPVIIHITDMNTEPIAAYHFDL
SGHAFGAMAKEGKDEELRKAGIIDMQFRRVRCKYPGETKVTFHVEKGSNPNYFAVLVKYVGGDG
DVVKVELKEKGSEEWKPLNESWGAIWRIDTPKPLKGPFSLRVTTESDQKLVANDVIPDNWKANAL
YKSEIQVD

>OsEXPB11 (UniProtKB accession number Q6H676)

MAKSCTLVLLLVALVGLSLLVSPIACSRKLSKPKPKPKPSMKKPVVRAHNNYTGSPSVTVTTGWAA
AGATYYGAPNGDGSDGGACGYQTAVGQRPFSSMIAAGSPSLYKGGKGCGACYEVKCTTNAACSG
QPATVVITDECPGGICLAGAAHFDMSGTSMGAMAKPGMADKLRAAGILQVQYRRVPCKYSGVNI
AFRVDQGANPFYFEVLIEFEDGDGDLNAVDLMEAGCGWTPMVQNWGALWRYNSNTGKALKAPF
SLRLTSDSGKVLVANNVIPASWKPGVTYRSLVNYS

>OsEXPB12 (UniProtKB accession number Q10G40)

MAAFEPHRLQLLYFIAITVLASVFQPCTSIELHRELSGWSNGIATWYGDPNGAGSEGGACGYQYAV
DQPPFSSRIAAGSPYIYDSGKGCGSCYRVVCAGNEACSGIPVTVVITDQGPGGPCLEELVDGQCMN
EAAHFDMSGTAFGAMARPGQADQLRGAGLLQIQYTRVECEWTGVGLTFVVDSGSNPNYLALLVE
YDDNDSDLAAVDIMPIGAGASGSWIPMQQSWGAVWRLNSGSALQGPFSVRLTFSSGQMFVASNAI
PAGWNPGMAYQPGGVAMRVRGRNGGRRGYEAVGMLGGLCHLLLLLLLMLFEL

>OsEXPB13 (UniProtKB accession number Q946J4)

MASSSLLLASVVVAAMVSAVSCGPPKVPPGPNITASYGDKWLEARATWYGAAKGAGRKDNSGAC
GYKDVDKAPFLGMNSCGNDPIFKDGKGCGSCFEIKCSKPKACSDKPVLIHVTDMNDEPIAAYHFD
LFGLAFGAMAKDGKDEELRKAGIIDT*FRRFKCKYPADTKITFHIEKASNPNYLALLVKYVAGDGD
VVEVEIKEKGSEEWKALKESWGAIWRIDTPKPLKGPFSVRVTTEGGEKIIAEDAIPDGWKADSVYK

SNVQAK*

>OsEXPB14 (UniProtKB accession number Q6H677)

MALAAKLLPSIVAFVALACCVLRSSVASVDHHRKLSGWSIGGATWYGPANGSGTDGGACGYQGD
VGQPPFNSMIAAGSPSIYESGKGCGSCYQVKCSGNPSCSGKPVTVVLTDLCPGGACLEEPVHFDLS
GTAFGAMAKPGQDDQLRNAGKLPVQYARVPCKWQGVDIAFRVDAGSNQYYLAVLVEDEDGDGD
LSAVDLMQSGGSGGGGSWAAMQQSWGAVWKYNSGPAPLQAPMSIRLTSGSGRTLVASNVIPAGW
QPGGTYRSIVNFRRED

>OsEXPB15 (UniProtKB accession number Q7XT40)

MASRFQLILSTFVVIAAVTMLPRPCASIEFHRKLSSWSNGGATWYGAANGAGSDGGACGYQGAVF
QAPFSSMIAAGSPSIYKSGLGCGSCYQVKCTGNSACSGNPVTVVLTDECPGGPCLSEPVHFDLSGTA
FGAMANPGQADQLRAAGVLQIQYNRVPCNWGGVKLTFVVDVGSNPNYFAVLVKYENGDGDLSG
VELMQTGAGAAWTQMQQSWGAVWKLNAGSALQAPFSIRLTSSSGKTLVASNVIPSGWKPGMSYIS
TVNF

>OsEXPB16 (UniProtKB accession number Q0DZ85)

MLIRSVLFVSLLSAAFVFDSGEAGAAHRVVDPEWHPATATWYGSADGDGSDGGACGYGTLVDVV
PMKTRVGAVSPVLFKGGEGCGACYKVRCLDASICSRRAVTVIVTDECPGGVCAFGRTHFDLSGAAF
ARLAVAGHGGQLQNRGEISVVYRRTACKYGGKNIAFHVNEGSTTFWLSLLVEFEDGDGDIGSMQL
KQANSAQWQDMKHIWGATWSLTPGPLVGPFSVRLTTLTTRQTLSAQDVIPKNWTPKATYTSRLNF
A

>OsEXPB17 (UniProtKB accession number Q7X6J9)

MAAASSRSFSLCVLLLLLLLAPPISASFLFDGGKSKSAAAAAAVDMEWRPATATWYGDAEGDGST
GGACGYGSLVDVVPMKARVGSVSPVLFKDGEGCGACYKVKCLDHGICSRRAVTVIVTDECPGGL
CAFGRTHFDLSGAAFSRMAVAGAGGHLRDRGQLSVVYRRTACKYGGKNIAFRVNEGSTNFWLSLL
VEFEDGQGDIGSMQIKQANSVEWLDMKHVWGATWCLVRGPLVGPFSVRLTTLSAQKALTARDVIP
RNWKPTATYTSRLNFEAAL

>OsEXPB18 (UniProtKB accession number Q5W6Z9)

MNSKFQLILSTFVVIAAFTLLPRPCASIEFHRKLSSWSNGGATWYGAANGAGSDGGACGYQAAVD
QAPFSSMIAAGSPSIYKSGLGCGSCYQVKCSGNSACSGNPVTVVLTDECPGGPCLSEPVHFDLSGTA
FGAMANPGQADQLRAAGVLQIQYNRVPCNWGGVMLTFAVDAGSNPSYFAVLVKYENGDGDLSG
MDLMQTGAGAAWTPMQQSWGAVWKLSAGAALQAPLSIRLTSSSGKTLVASNVIPSGWKPGASYT
STVNY

>OsEXLA1 (UniProtKB accession number Q10S70)

MAVSVRCCFGSSSLSHHARLLLVIVALLAPRLASGCDRCVRRSRAAYYTSSLTLTAGSCGYGTAAAT
FNGGGFLAAAGPALYRGGVGCGACYQVRCKDKKLCSNAGARVVVTDRARTNRTGLVLSSPAFAA
MARPGMAASLTELAAVDVEYKRVPCEYRHRSLSVRVDERSRGPNELTISFLYQGGQTDIVAVDVAQ
VGSSSWKFMTREHGPSWSMANAPPGPLQMRLVVTGGYDGKWVWADREVLPRRWRAGEVYDTG
VQITDIAQEGCFPCDTHEWK

>OsEXLA2 (UniProtKB accession number Q7XCL0)

MAVRCCSSMASASVVLFFVVVGMSASMVSGCDRCVRRSKAGFRDSSIALNAGSCGYGSLAASFN
GGHLAAASPALFRGGVGCGACFQVRCKDGKLCSTAGAKVVVTDEARSTNRTDLVLSAAAYAAMA
RPGMAAQLRTRRAVDVEYKRVPCEYAAGRNLSIRVEEKSRPPRELSIRFLYQGGQTDIVAVDVATVG
SSNWKFMTRDYGPAWSTAQAPAGPLQFRVVVTGGYDGKWVWADGEVLPRRWTAGRVYDAGVQI
ADVAQEGCYPCDTQEWK

>OsEXLA3 (UniProtKB accession number Q8H274)

MAVLLSILSSSFLLLLAASSSSTPRASACERCVRNGKAAYSPSLSPLPPGGGGGCGYGAMAMEMEL
NGGFLAAGGPRQHRGGLGCGRCFQMRCRNAEVCSNAGVRVVLTDFHRSNSTDFLLGGPAFAGLA

KPGMAHKLKKLDALSVEYRRIPCDYKDKNLSILVEEQSKRPNNLVIKFLYQGGQTDILAVDVAQVG
SSDWRFMTRVYGPVWSIDRAPNGPLQFRAVVTGGYDGKWVWADREVLPANWQPGQVYDTGARI
ADVARESCLDCATLDWK

>OsEXLA4 (UniProtKB accession number Q5Z980)

MDDNGDVHFCHRATAVVALLLLHLVVVANAAAHSCDWCTPRHSTVSILPTPTHAAHLTGGACGFG
AAPMELNVAAVTADLFRHGHACGACYQLRCRDRRLCGEDGVKVVVADMAKQPEQEGEMNRTAG
GSLQFRITEDAFAAMAKQGVSAHELTRQRTLEVDFRRIPCEYRESRRLAVRVEEASRNPTHLAIRFL
YQGGQTDIAAVEIAQANATPPSSSYYSSWRYMTRRDGAPGVWTTSRAPVGPLRLRVVVTAGSGGK
WLRSDGEVLPADWRPGEVYDTGLRVTDVAVRSCSLSCAIQDMDSDDGEEEELR

>OsEXLB1 (UniProtKB accession number Q850K7)

MAQLLRRHLPVILSLILFLSKATADANFTVSRAAYYPNSDIKGTENGACEYGAFGATLNNGDVSAS
ASLYRDGVGCGACYQVRCTNPYYCSPNGVTIVITDSGASDGTDFILSQHAFTRMAQSTDAGTALLT
LGVVGIEYRRVSCTYPNKNIVFKITESSNFPNYLEFEIWYQQGNQDIIAVQLCETVNLTCQLLSRTHG
AVWAAVSPPSGPLSIRMLFSSGAPRGGDTWLVPTNIVPQNWTAGATYDSGVQVQLQ

Text S3 Promoter and gene sequence of *HvEXPB7* in XZ5, XZ54 and Tadmor. Yellow represents RHEs in promoter; Red represents SNPs in CDS; green represents intron; pink represents start and stop codons.

XZ5 (GeneBank accession number KT000616)

TTCGCAAGTTTAAATCATCACAAGTTCAACATCACAACAAAAGTATCATCATAACAAGAAAAGC
AACAAGTCATGTAAAAGGCATAGAAGACCGTCAAGTTTGTCATCGCAAAGGCTTAAGCCACGT
CCACCACCATCGCCAAAGCCAAGATCACCAAAGCCGAGGTCACTACCGCTACCGTCACCGCCA
CACCTCGCTAGACCGGAGTGACTGGGCTACTTGACTCAGGAGTGGAACCATCATTAGCATCACC
ACCGGCTGATGATCCATCGGTTCCCTACATGTTTGAAAAGAGATGGTAAGGCGATATATGATTGT
ATTGACTAAACAATAAGTTGTGGTTGAATAGTTTGGTGATGTACTAACCCGGGTGAAATAGTGTT
GGGACACAAATTGTTCAAACGTAGGCAGCATAGCTTGTCGTCCAGGAGGTGGTGGTGGTGGTA
CCATTGTCGGAATCTGCCCGAATGCTATAGATGTAAACATAATCTTCGTCATGCGAGTGTTGTGC
TCCGTGGCTGCTGCAACTCTCTGGTTCCAGTCAAAGAGCTGCTGACATGGGAACTCCAAGTAG
ACCTACAATATGACATGATGGAACTGATAAACCACAACAATGCGAATAATAAGGGTCGAAAGAA
TGAAGATACAAGGGAAAATACTTACAGCGTGCTGCTTGGCTAGGATGGACTATGCACTGGATGT
ACTCGGGTGCTGGCATGACCTCGGCCTCGGTTTGGTAGCTCCGGTTCGTGTGTACGAGACAGA
AGGGATGATCACAGGAGACAGAAGGGATGATCACAGAATCCCGAACCGACTGCCGACCATGAC
TCTTCGGCCATATGCTGACCATCGCTATCTCGTTGGTATCCATAGACATGGGGTCTGATGTGCCA
GGATACCACTTCTAAAACAGCTCTGTGTAGGACGCCAGGTTCTCCTCGGTATCGCCGTAGTACT
GACCCCCACCAGCCTTGCGATTAGTCCACGCACGGGCGAGCCCCCACGACTCGATGTCTAAG
AGCGGCCGCTTCAATTTCTCCTCCTAGACTGCCAATTAGAGGTTTGTCACACAAAAGAACATAC
GGTAAATAGAAGATCAAGAATGTTTCATGTACATATATATACCTGAAGTTTCTTGTGATGCGAGT
GGTTCCGGTTTCCCTCACCGTGTGTTCCGTCAGATCCTCGATTAGCTTTGTTTGTGTTGCTCTTG
GCAGAAAACTTGATCCACAGATCCACCAATCTCGCCCATCCATCATTCTTTCCAAAGCACCAATT
AGGAGGCACCTACATAATGAATCATAATGGCATGTGAACACGAAACAATGAAATTTACCACAA
ACGTCGGAATGAAAAGTATGTTATACTTACCAACATGTACTGCTCCCTAGTCATGGTAAGGTTCT
GTGCCGCGCTTCTTCCTTGGCTATCTTCTCGCCCAGAACAGTGTGATAATACGTCCGGATGGCCA
CCCAAAGCACCTCGTATTGTATTTGTCAGGTCTGCTTCCTCGCAGCCCGCAGCACGAACTCATC
GGCCTTGTCCTTATGCTCCTGAAGAATTTCTATAGCATGGCAACCCTTTAGCCAGCACTGCAAAT
TCCGAGGATGAAAATTTTCACCATTTACCTTTTCTAGGCTTACGATTTTATATGTTGCAATCATGG
CAAAAATAAATGAAAGTACATGGCAATTACTGGGCTGCAACATGAAAATTTCCAGACAATTTTT
TTGTATAACATGACAACTTTTACTATACAACAGCAGAGACAGATCATGACGTGGCCGGAGAAA
AGTGGGTGGTTTGTTTGCAATGGCCAGATTGTGTAGATGTTCCAACAATTTGTACTAGTCTTGTT
CCATTGTATCTTTCGATGCTTCGTTATTTTGTCACCGGATGGATTTTAGGGTTGTATTATGCCGTG
TCATGGACTGACGGCCTCACGGGTAGCGTGTAACCAAAACCTTTATCAGATTATTTTAGCATGT
CCTGATCGGCTACTGATGCGCACGTAGCTTAAGATGGGTATACTAGTTTGGTATTTTGGAAGTAC
ATAGACACGCGAGCTAGCGCTCCACTGGTTACAGGACAAATCATGTGGCTTTATATGGTCGGCA
ATTTGCATCATATGCTGGCTGCCGGCATGTCATACACAGTACTTGTATGTAGAGTGTATATATATGT
GTATGGTGGATGATCCGTAAAAAAAAATGTATGGTTGATGATGGAAGATGTTTTTTAAAACATAA
AAGCAGAGAGAAATGCATCTCGTGACCACGGGCAAGGGTGTAAGCAGCCGGTTGAGTGCCTC
CATCCATGCACATGTGAACCGGTAGGGCCCAAGATTGCCAATCACCAGTCATCTCCATTCATTCC
TTCCTTCTTCCCTTTGATCTCCAGAAGCACCTCACTGGCTGGCCTATAAATTCGCAATCTCCCCA

ACGGCATTAACCACCAGAGTAGGGTCATCGATCAGTAACCAACGAGACCAGCAAGCGAGCCAT
GGCAGCGGCCTTTTCCTCCTACTCCGTCGCGGCCGCTTTCCTCTGCCTGCTCGCCGCCAACGGC
TGCTGCGGCTGCCCCTGGTTTCTCCCTGCTACATTCTGCCCTGAACCGACACCCGACCCCATAC
CTACACCTGACCCCGCTCCAGCTACACCTCCGCCTGCTCTGGCTACCCCCAGCCCAGGCTCCGG
CTCTGGCAGCGCCAACGGTTCCATGGGCGGCTGGCTGGACGCCAGGGCCACATGGTACGGCGC
TCCGGATGGCGCCGGGCCGTTGGACAACGGTGGCGCGTGCGGGTTCAAGAACGTGAACCTGC
CGCCCTTCAACGCCATGACGTCGTGCGGCAACGAGCCGATCTTCAAGGACGGCAAGGGATGCG
GCTCCTGCTACCAGGTCAGCCGTTTAATTATTTGCTTGACATTGACAGTACGTGCTTGTCGTTCC
GTTTATCCAGCTCCAAACTGATGCATTCATTGTTGCACGCAGATAAGGTGCGTGGGCAAGGTTC
ACCCAGCGTGCTCCGGCGACCCCGAGACGGTGATCATCACCGACATGAACTACTACCCGGTCG
CCCGCTACCACTTCGACCTCAGCGGCACTGCCTTCGGCGCCATGGCCAAGGACGGCCGCAACG
ACGAGCTCCGCCACGCCGGCATCATCGACATGCAGTTCAAGAGGGTGCCGTGCCAGTACACAG
GGCTGTCCGTGACGTTTCACGTGGAGAAGGGGTCGAACCCGAATTACCTGGCGATCCTGGTGG
AGTACGGGAACGGCGACGGCGACGTGGCGCAGGTGGACCTCATGGACGGCGGCGAGCCAACG
GTGTCCTGGAGGCCGATGAGGGAGTCCTGGGGCTCCATCTGGCGCCTGGACCACGCGGCGCCCG
CTTCGGGGGCCCTTCTCGCTGCGGGTCACCAACGGGTCCGGCAGGTCGCTCGTCGCGGACCAG
GTCATCCCCGCCGACTGGCAGCCCGACACCGTGTACAGCTCCGACGTCCAGTTCGATGAATGA

XZ54 (GeneBank accession number KT000617)

TTCGCAAGTTTAACATCATCACAAGTTCAACATCACAACAAAAGTATCATCATAACAAGAAAAG
CAACAAGTCATGTAAAAGGCATAGAAGACCGTCAAGTTTGTCATCGCAAAGGC**T**TAAGC**CACG**
TCCACCACCATCGCCAAAGCCAAGATCACCAAAGCCGAGGTCACTACCGCTACCGTCACCGCC
ACACCTCGCTAGACCGGAGTGACTGGGCTACTTGACTCAGGAGTGGAACCATCATTAGCATCA
CCACCGGCTGATGATCCATCGATTCCCTACATGTTTGAAAAGAGATGGTAAGGCGATATATGATT
GTATTGACTAAACAATAAGTTGTGGTTGAATAGTTTGGTGATGTACTAACCCGGGTGAAATAGTG
TTGGGCCACAAATTGTTCAAACGTAGGCAGCATAGCTTGTCGTCCAGGAGGTGGTGGTGGTGG
TACCATTGTCGGAATCTGCCTGAATGCTATAGATGTAAACATAATCTTCGTCATGCGAGTGTTGT
GCTCCGTGGCTGCTGCAACTCTCTGGTTCCAGTCAAAGAGCTGCTGACGTGCGAACTCCAAGT
AGACCTACAATATGACATGATGGAACTGATAAACCACAACAATGCGAACAATAAGGGTCGAAA
GAATGAAGATACAAGGGAAAATACTTACAGCGTGCTGCTTGGCTAGGATGGACTATGCACTGGA
CGTACTCGGGTGCTGGCATGACCTCGGCCTCGGTTTGGTAGCTCCGGTT**CGTG**TGTACG**A**GAC
AGAAGGGATGATCACAGGAGACAGAAGGGATGATCACAGAATCCCGAACCGACTGCCGACCA
TGACTCTTCGGCCATATGCTGACCATCGCTATCTCGTTGGTATCCATAGACATGGGATCTGATGTG
CCAGGATACCACTTCTAAAACAGCTCTATGTAGGACGCCAGGTTCTCCTCGGTATCGCCATAGTA
CTGACCCCCACCAGCCTTGCGATTAG**T**CCGCG**CACG**GGCGAGCCCCCACGACTCGATGTCTAA
GAGCGGCCGCTTCAATTTCTCCTCCTAGACTGCCAATTAGAGGTTTGTCACACAAAAGAACATA
CGGTAAATAGAAGATCAAGAATGTTTCATGTACATATATATACCTGAAGTTTCTTGTGATGCGAG
TGGTTCCGGTTTCCCTCACCGTGTGTTCCGTCAGATCCTCGATTAGCCTTGTTTGTGTTGCTCTT
GGCAGAAAACTTGATCCACAGATCCACCAATCTCGCCCATCCATCATTCTTTCCAAAGCACCAA
TTAGGAGGCACCTACATAATGAATCATAATGGCA**TG**TGAA**CACGA**AACAATGAAATTTACCACA
AACGTCGGAATGAAAAGTATGTTATACTTACCAACATGTACTGCTCCCTAGTCATGGTAAGGTTC
TGTGCCGCGCTTCTTCCTTGGCTATCTTCTCGCCCAAAACAGTGTGATAATACGTCCGGATGGCC
ACCCAAAGCACCTCGTATTGTATTTGTCAGGTCTGCTTCCTCGCAGCCCGCAGCACGAACTCAT
CGGCCTTGTCCTTATGCTCCTGAAGAATTTCTATAGCATGGCAACCCTTTAGCCAGCACTGCAAA
TTCCGAGGATGAAAATTTTCACCATTTACCTTTTCTAGGCTTACGATTTTATATGTTGCAATCATG
GCAAAAATAAATGAAAGTACATGGCAATTACTGGGCTGCAACATGAAAATTTCCTGACAATTTT
TTTGTATAACATGACAACTTTTACTATACAACAGCAGAGACAGATCATGA**CGTG**GCCGG**A**GAAA

AGTGGGTGGTTTGTTTGCAATGGCCAGATTGTGTAGATGTTCCAACAATTTGTACTAGTCTTGTT
CCATTGTATCTTTCGATGCTTCGTTATTTTGTCACCGGATGGATTTTAGGGTTGTATTATGCCGTG
TCATGGACTGACGGCCTCACGGGTAGGGTGTAACCAAAACCTTTATCAGATTATTTTAGCATGTC
CTGATCGGCTACTGATGCGCACGTAGCTTAAGATGGGTATACTAGTTTGGTATTTTGGAAGTACA
TAGACACGCGAGCTAGCGCTCCACTGGTTACATGACAAATCATGTGGCTTTATATGGTCGGCAAT
TTGCATCATATGCTGGCTGCCGGCATGTCATACACAGTACTTGTATGTAGAGTGTATATATATATGT
ATGGTGGATGATCCGTAAAAAAAAATGTATGGTTGATGATGGAAGATGTTTTTTAAAACATAAA
AGCAGAGAGAAATGCATCTCGTGACCACGGGCAAGGGTGTAAGCAGCCGGTTGAGTGCCTCC
ATCCATGCACATGTGAACCGGTAGGGCCTTGATGCCAATCACCAGTCATCTCCATTCATTCCTTC
CTTCTTCCTTTTGATCTCCAGAAGCACCTGAGTGGGTGGCCTATAAATTCGCAATCTCCCCGACG
GCATTAACCATCAAAGTAGGGTCATCGATCAGTAACCAACGAGACCACAAGCGAGCCATGGCA
GCGACCTTTTCCTCCTACGCGATCGCGGTCGCTTTCCTCTGCCTGCTCGCCGCCAACGGCTGCA
GCGGCTGCCCCTGGTTTCTCCCTGCTACATTCTGCCCTGAACCTACACCCGACCCCATACCTACA
CCAAACCCCGCTCCAGCTACACCTCCGCCTGCTCCGGCGACCACCAGCCCAGGCTCCGGCGCT
GGCAGCACCAATGGTTCCACCGGCGGCTGGCTGGACGCCAGGGCCACATGGTACGGCGCTCCG
GATGGCGCCGGGCCGTTGGACAACGGTGGCGCGTGCGGGTTCAAGAACGTGAACCTGCCGCC
CTTCAACGCCATGACGTCGTGCGGCAACGAGCCGATCTTCAAGGACGGCAAGGGATGCGGCTC
CTGCTACCAGGTCAGCCGTTTAATTATTTGCTTGACATTGACAGTACGTGCTTGTCGTTCCGTTT
ATCCAGCTCCAAACTGATGCATTCATTGTTGCACGCAGATAAGGTGCGTGGGCAAGGTTCACCC
AGCGTGCTCCGGCGACCCCGAGACGGTGATCATCACCGACATGAACTACTACCCGGTCGCCCG
CTACCACTTCGACCTCAGCGGCACTGCCTTCGGCGCCATGGCCAAGGACGGCCGCAACGACGA
GCTCCGCCACGCCGGCATCATCGACATGCAGTTCAAGAGGGTGCCGTGCCAGTACACAGGGCT
GTCCGTGACGTTTCACGTGGAGAAGGGGTCGAACCCGAATTACCTGGCGATCCTGGTGGAGTA
CGGGAACGGCGACGGCGACGTGGCGCAGGTGGACCTCATGGACGGCGGCGAGCCAACGGTGT
CCTGGAGGCCGATGAGGGAGTCCTGGGGCTCCATCTGGCGCCTGGACACGCGGCGCCCGCTTC
GGGGGCCCTTCTCCCTGCGGGTCACCAACGGGTCCGGCAGGTCGCTCGTCGCCGACCAGGTCA
TCCCCGCCGACTGGCAGCCCGACACCGTGTACAGCTCCGACGTCCAGTTCGATGAATGA

Tadmor (GeneBank accession number KT000618)

TTCGCAAGTTTAACATCATCACAAGTTCAACATCACAACAAAAGTATCATCATAACAAGAAAAG
CAACAAGTCATGTAAAAGGCATAGAAGACCGTCAAGTTTGTCATCGCAAAGGCTTAAGCCACG
TCCACCACCATCGCCAAAGCCAAGATCACGAAAGCCGAGGTCACTACCGCTACCGTCACCGCC
ACACCTCGCTAGACCGGAGTGACTGGGCTACTTGACTCAGGAGTGGAACCATCATTAGCATCA
CCACCGGTTGATGATCCATCGGTTCCCTACATGTTTGAAAAGAGATGGTAAGGCGATATATGATT
GTATTGACTAAACAATAAGTTGTGGTTGAATAGTTTGGTGATGTACTAACCCGGGTCAAATAGCG
TTGGGCCACAAATTGTTCAAACGTAGGCAGCATAGCTTGTCGTCCAGGAGGTGGTGGTGGTGG
TACCATTGTCGGAATCTGCCCGAATGCTATAGATGTAAACATAATCTTCGTCATGCGAGTGTTGT
GCTCCGTGGCTGCTGCAACTCTCTGGTTCCAGTCAAAGAGCTGCTGACGTGCGAACTCCAAGT
AGACCTACAATATGACATGATGGAACTGATAAACCACAACAATGCGAACAATAAGGGTCGAAA
GAATGAACAACAAGGGAAAATACTTACAGCGTGCTGCTTGGCTAGGATGGACTATGCACTGGA
CGTACTCGGGTGCTGGCATGACCTCGGCCTCGGTTTGGTAGCTCCGGTTCGTGTGTACGAGAC
AGAAGGGATGATCACAGGAGACAGAAGGGATGATCACAGAATCCCGAACCAACTGCCGACCA
TGACTCTTCGGCCATATGCTGACCATCGCTATCTCGTTGGTATCCATAGACATGGGGTCTGATGT
GCCAGGATACCACTTCTAAAACAGCTCTGTGTAGGACGCCAGGTTCTCCTCGGTATCGCCGTAG
TACTGACCCCCACCAGCCTTGCGATTAGTCCGCGCACGGGCGAGCCCCCACGACTCGATGTCT
AAGAGCGGCCGCTTCAATTTCTCCTCCTAGACTGCCAATTAGAGGTTTGTCACACAAAAGAACA
TACGGTAAATAGAAGATCAAGAATGTTTCATGTACATACATATACCTGAAGTTTCTTGTGATGCG

AGTGGTTCCGGTTTCCCTCACCGTGTGTTCCGTCAGATCCTCGATTAGCCTTGTTTGTGTTGCTC
TTGGCAGAAAACTTGATCCACAGATCCACCAATCTCGTCCATCCATCATTCTTTCCAAAGCACC
AATTAGGAGGCACCTACATAATGAATCATAATGGCA**TG**TGAA**CACGA**AACAATGAAATTTACCA
CAAACGTCGGAATGAAAAGTATGTTATACTTACCAACATGTACTGCTCCCTAGTCATGGTAAGGT
TCTGTGCCGCGCTTCTTCCTTGGCTATCTTCTCGCCCAGAACAGTGTGATAATACGTCCGGATGG
CCACCCAAAGCACCTCGTATTGTATTTGTCAGGTCTGCTTCCTCGCATCCCGCAGCACGAACTC
ATCGGCCTTGTCCTTATGCTCCTGAAGAATTTCTATAGCATGGCAACCCTTTAGCCAGCACTGCA
AATTCCGAGGATGACAATTTTCACCATTTACCTTTTCTAGGCTTACGATTTTATATGTTGCAATCA
TGGCAAAAATAAATGAAAGAACATGGCAATTACTGGGCTGCAACATGAAAATTTCCTGACAATT
TTTTTGTATAACATGACAACTTTTACTATACAACAGCAGAGACAGATCATGA**CGTG**GCCGG**A**GA
AAAGTGGGTGGTTTGTTTGCAATGGCCAGATTGTGTAGATGTTCCAACAATTTGTACTAGTCTTG
TTCCATTGTATCTTTCGATGCTTCGTTATTTTGTCACCGGATGGATTCTAGGGTTGTATTATGC**CG**
TGTCATGG**A**CTGACGGCCTCACGGGTAG**CGTG**TAAC**CA**AAACCTCTATCACATTATCTTAGCAT
GTCCTGATCGGCTAC**TG**ATGCG**CACG**TAGCTTAAGATGGGTATACTAGTTTGGTATTTTGGAAGT
ACATAGACACGCGAGCTAGCGCTCCACTGGTTACATGACAAATCATGTGGCTTTATATGGTCGG
CAATCTGCATCATATGCTGGCTGCCGGCATGTCATACACAGTACTTGTATGTAGAGTGTATATATA
TATGTATGGTGGATGATCCGTAAAAAAAATGTATGGTTGATGATGGAAGATGTTTTTTAAAACAT
AAAAGCAGAGAGAAATGCATC**T**CGTGAC**CACG**GGCAAGGGTGTAAGCAGCCGGTTGAGTGCC
TCCATCCATGCACATGTGAACCGGTAGGGCCTTGATGCCAATCACCAGTCATCTCCATTCATTCC
TTCCTTCTTCCTTTTGATCTCCAGAAGCACCTGAGTGGGTGGCCTATAAATTCGCAATCTCCCCG
ACGGCATTAACCATCAGAGTAGGGTCATCGATCAGTAACCAACGAGACCAGCAAGCGAGCCAT
GGCAGCGACCTTTTCCTCCTACGCGATCGCGGTCGCTTTCCTCTGCCTGCTCGCCGCCAACGGC
TGCAGCGGCTGCCCCTGGTTTCTCCCTGCTACATTCTGCCCTGAACCTACACCCGACCCCATAC
CTACACCAAACCCCGCTCCAGCTACACCTCCGCCTGCTCCGACGACCACCAGCCCAGGCTCCG
GCGCTGGCAGCACCAATGGTTCCACCGGCGGCTGGCTGGACGCCAGGACCACATGGTACGGCG
CTCCGGATGGCGCCGGGCCGTTGGACAACGGTGGCGCGTGCGGGTTCAAGAACGTGAACCTG
CCGCCCTTCAACGCCATGACGTCGTGCGGCAACGAGCCGATCTTCAAGGACGGCAAGGGATGC
GGCTCCTGCTACCAGGTCAGCCGTTTAATTATTTGCTTGACATTGACAGTACGTGCTTGTCGTTC
CGTTTATCCAGCTCCAAACTGATGCATTCATTGTTGCACGCAGATAAGGTGCGTGGGCAAGGTT
CACCCAGCGTGCTCCGGCGACCCCGAGACGGTGATCATCACCGACATGAACTACTACCCGGTC
GCCCGCTACCACTTCGACCTCAGCGGCACTGCCTTCGGCGCCATGGCCAAGGACGGCCGCAAC
GACGAGCTCCGCCACGCCGGCATCATCGACATGCAGTTCAAGAGGGTGCCGTGCCAGTACACA
GGGCTGTCCGTGACGTTTCACGTGGAGAAGGGGTCGAACCCGAATTACCTGGCGATCCTGGTG
GAGTACGGGAACGGCGACGGCGACGTGGCGCAGGTGGACCTCATGGACGGCGGCGAGCCAAC
GGTGTCCTGGAGGCCGATGAGGGAGTCCTGGGGCTCCATCTGGCGCCTGGAGACGCGGCGCCC
GCTTCGGGGGCCCTTCTCGCTGCGGGTCACCAACGGGTCCGGCAGGTCGCTCGTCGCCGACCA
GGTCATCCCCGCCGACTGGCAGCCCGACACCGTGTACAGCTCCGACGTCCAGTTCGATGAATG
A
